# Membrane and soluble VTCN1 (B7H4) converge on Src signaling to mediate gemcitabine resistance in intrahepatic cholangiocarcinoma

**DOI:** 10.7150/thno.130394

**Published:** 2026-08-24

**Authors:** Yi-Ru Pan, Sheng-Hsuan Lin, Yu-Chan Chang, Shih-Ming Jung, Chiao-En Wu, Wen-Kuan Huang, Chun-Nan Yeh

**Affiliations:** 1 Department of Surgery, Chang Gung Memorial Hospital, Linkou, Chang Gung University, Taoyuan 333, Taiwan.; 2 Department of Biomedical Imaging and Radiological Sciences, National Yang Ming Chiao Tung University, Taipei 112, Taiwan.; 3 Department of Pathology, Chang Gung Memorial Hospital, Linkou, Taoyuan 333, Taiwan.; 4 Division of Hematology-Oncology, Department of Internal Medicine, Chang Gung Memorial Hospital, Linkou, Chang Gung University College of Medicine, Taoyuan 333, Taiwan.; 5 Division of Hematology-Oncology, Department of Internal Medicine, New Taipei Municipal TuCheng Hospital, New Taipei City, Taiwan.; 6 Institute of Stem Cell and Translational Cancer Research, Chang Gung Memorial Hospital, Linkou, Taoyuan 333, Taiwan.; 7 School of Medicine, National Tsing Hua University, Hsinchu 30013, Taiwan.

**Keywords:** intrahepatic cholangiocarcinoma, B7H4, soluble VTCN1, gemcitabine resistance, Src.

## Abstract

**Rationale:**

A combination of gemcitabine (GEM)-based chemotherapy and an immune checkpoint inhibitor is the standard treatment for patients with advanced intrahepatic cholangiocarcinoma (iCCA). However, 70% of patients develop progressive disease following GEM treatment, highlighting the urgent need for therapeutic strategies targeting GEM-resistant (GR) disease.

**Methods:**

We established GR iCCA sublines and identified 9 upregulated genes by RNA-sequencing and cDNA microarray. Among the 9 genes, we demonstrated that VTCN1 expression improved GR. The enhanced effect of VTCN1 on GR was validated in a spontaneous rat iCCA model, a xenograft mouse model, an orthotopic mouse model, and iCCA specimens. A phospho-kinase array was used to identify the downstream effector of membrane and soluble VTCN1 (sVTCN1).

**Results:**

VTCN1 expression was significantly elevated in GEM non-responders and independently predicted poor progression-free survival (HR = 2.038, P = 0.035). Plasma sVTCN1 levels correlated with tumor VTCN1 expression (*r* = 0.8752, P = 0.002) and tumor burden (*r* = 0.7696, P < 0.0001). Mechanistically, membrane-bound VTCN1 directly interacted with integrin β1 to enhance FAK phosphorylation, while sVTCN1 bound EGFR to create a positive feedback loop increasing EGFR phosphorylation. Both pathways converged on Src family kinase activation (Y419 phosphorylation) to drive GR. The VTCN1-Src axis was associated with upregulation of cytosolic 5'-nucleotidase 3 (NT5C3) expression to promote GEM resistance. VTCN1 depletion, Src depletion, or Src inhibition with dasatinib restored GEM sensitivity. Combining VTCN1 antibody or dasatinib with GEM significantly suppressed resistant tumor growth and enhanced CD8^+^ T cell infiltration *in vivo*.

**Conclusions:**

Membrane and soluble VTCN1 drive GR through dual pathways converging on Src activation. VTCN1 represents both a prognostic biomarker and therapeutic target, with Src inhibition providing a rational strategy to overcome chemotherapy resistance in iCCA.

## Introduction

VTCN1 (V-set domain containing T cell activation inhibitor 1, B7H4), a member of the B7 family, is aberrantly expressed in inflammation, autoimmune diseases, and tumors 1. VTCN1 protein has 282 amino acids, including an amino-terminal extracellular domain, a large hydrophobic trans-membrane domain, and a very short intracellular domain of just two amino acids 2. It is rarely detected in normal tissues 3. As a co-inhibitory molecule, VTCN1 suppresses T cell immune response and promotes immune escape 4. Previous studies have linked VTCN1 to cancer progression, investigating the role in proliferation and migration *in vitro* and tumorigenesis *in vivo* in hepatocellular carcinoma 5. VTCN1 knockdown increases doxorubicin sensitivity via the PTEN/PI3K/AKT pathway in triple-negative breast cancer 6. A soluble form of VTCN1 (sVTCN1) circulates systemically and has roles in cancers, inflammation, autoimmunity, and pregnancy 1. Notably, serum VTCN1 combined with CEA significantly improves the sensitivity and specificity of diagnosis for colorectal cancer and malignant pleural effusion 7, 8. Bile sVTCN1 levels can also facilitate early diagnosis of extrahepatic CCA patients 9.

Cholangiocarcinoma (CCA), a malignant tumor from cholangiocytes, is classified into three subtypes by anatomic location, including intrahepatic CCA (iCCA), perihilar CCA (pCCA), and distal CCA (dCCA) 10. iCCA, the second most common primary hepatic malignancy, is often diagnosed at an advanced stage with a poor prognosis 11. For patients with advanced-stage or unresectable iCCA, a combination of gemcitabine (GEM)-based chemotherapy and an immune checkpoint inhibitor remains the standard of care 12. Recently, TOPAZ-1 and KEYNOTE-966 studies show positive results that durvalumab or pembrolizumab plus chemotherapy demonstrates longer survivals than those treated with traditional chemotherapy, respectively 12, 13. However, primary or acquired GEM resistance (GR) significantly limits therapeutic efficacy 14, with objective response rates of only 25-30% and a median progression-free survival of 5-7 months 13. Thus, it is a critical challenge to overcome GR for patients with advanced or unresectable iCCA.

Gemcitabine (dFdC) is the most widely used pyrimidine analogue. GEM is transported into cells by hCNTs (concentrative nucleoside transporters) or hENTs (equilibrative nucleoside transporters) 15, 16. Once GEM is transported into cells by transporters. GEM is metabolized to dFdCTP 17. The dFdCTP incorporates into the DNA strand, resulting in the inhibition of DNA synthesis. The dysregulation of the proteins participating in GEM metabolic pathways causes GEM resistance in several cancers 18.

In immunosuppressive functions, VTCN1 suppresses T cell activation, proliferation, and cytokine secretion 19. In tumors, serum sVTCN1 correlates with the progression in several malignancies 20-22. However, VTCN1-mediated GR in iCCA is poorly understood. In this research, we uncovered that VTCN1 plays a critical role in GR in iCCA. Mechanistically, membrane-bound VTCN1 was associated with integrin β1, enhancing focal adhesion kinase (FAK) recruitment and phosphorylation. Additionally, sVTCN1 created a positive feedback loop by binding to and promoting EGFR phosphorylation in iCCA cells. Both interactions promoted Src phosphorylation, ultimately driving GEM insensitivity in iCCA. Importantly, Src inhibition with dasatinib restored sensitivity in resistant models, providing a rationale for clinical investigation.

## Materials and Methods

### Intrahepatic cholangiocarcinoma patient samples and immunohistochemistry (IHC)

This study included 72 patients with pathologically confirmed iCCA who received GEM-based chemotherapy at Linkou Chang Gung Memorial Hospital between 2013-2017 (Figure 2A-D; Table 1; Table S1). Inclusion criteria were histologically confirmed iCCA, received at least one cycle of GEM-based chemotherapy, available tumor tissues for IHC, and complete clinical follow-up data. Exclusion criteria were mixed hepatocellular-cholangio-carcinoma, concurrent other malignancies, insufficient tissue for analysis. Treatment response was assessed according to RECIST v1.1 criteria: partial response (PR, n = 10), stable disease (SD, n = 32), or progressive disease (PD, n = 30). For plasma sVTCN1 analysis (Figure 2E-H; Figure S2D), blood samples were collected from patients with gallstones or iCCA. The study was approved by the Institutional Review Board of Linkou Chang Gung Memorial Hospital (IRB201900137B0, 202101668B0, 202302052B0, and 202201547B0). All patients provided written informed consent. Immunohistochemistry was performed as previously described 23. In brief, sections were incubated with VTCN1 antibody (Table S5) at 4 °C overnight, and the images were detected by the Dako REAL EnVision Detection System (K500711; Agilent Technologies, Inc., Santa Clara, CA, USA). We calculated the H-scores by multiplying the staining intensity by the percentage of positive cells. For detecting sVTCN1 in the patient's blood, plasma samples were prepared as previously described 24, and the levels of soluble VTCN1 were determined using a Human B7H4 DuoSet ELISA kit (DY6576-05; R&D Systems, Minneapolis, MN).

### Rat and mouse experiments

For the TAA-induced iCCA rat experiments (IACUC2019011601) in Figure 7E-F, the 10-week Sprague-Dawley rats were fed drinking water with TAA (thioacetamide, 300 mg/L) for 30-35 weeks 25. The spontaneous iCCA was detected by an animal positron emission tomography (PET) system. The tumor-bearing rats received low-dose GEM (25 mg/kg) weekly by intraperitoneal injection for 8 weeks and then received full-dose GEM (50 mg/kg) weekly by intraperitoneal injection, 200 μg of an isotype control (MAB1050; R&D Systems, Minneapolis, MN) or VTCN1 antibody (MAB21542; R&D Systems, Minneapolis, MN) three times a week by intravenous injection*, or* control PBS for another four weeks. The iCCA tumors were confirmed by animal PET every two weeks. ¹⁸F-FDG PET/CT imaging was performed using a Mediso nanoScan PET/CT scanner (Mediso Ltd., Budapest, Hungary). The animals were fasted overnight for 8 h before 18.5 MBq (0.5 mCi) ¹⁸F-FDG radiotracer injection via the tail vein. Animals were anesthetized and scanned 90 min post-tracer injection. A 30 min period for the static protocol was used. For detection of CD8^+^ T cells, a section was incubated with CD8 antibody (Table S5) at 4 °C overnight. The number of CD8^+^ T cells represents the averages from four 0.33 mm^2^ random fields. For the xenograft mouse experiments (IACUC2021092702) in Figure S7E-G, 7 x 10^6^ HuCCT1-GR cells were subcutaneously injected into BALB/c nude mice. Once the tumor volumes reached 100-150 mm³, the mice were injected intraperitoneally with 50 mg/kg GEM once per week, administered 20 mg/kg dasatinib (Dasa) via oral gavage five times per week, or the control solvent for 25 days. For the orthotopic mouse experiments in (IACUC2026012301) in Figure 7G-I, 2.5 x 10^5^ mouse iCCA ICKP cells 26 overexpressing VTCN1 or a control vector were injected into the left lobes of the livers of C57BL/6J mice. The mice were treated for 2 weeks with either GEM alone (25 mg/kg, intraperitoneally, twice weekly) or a combination of GEM and dasatinib (30 mg/kg, oral gavage, four times weekly. Tumor growth was evaluated by AMI HTX imaging system (Spectral Instruments Imaging, LLC, Tucson, AZ, USA) twice a week for 20 days. To analyze the tumor microenvironment, tumors were collected by enzymatic digestion, and the cells were then stained with antibodies (Table S5) according to the manufacturer’s protocols. Data were acquired by BD LSRFortessa (New Jersey, USA) and analyzed using FlowJo software. All animal experiments were performed in accordance with the institutional animal welfare guidelines of Chang Gung Memorial Hospital (CGMH). Sprague-Dawley rats, BCg-Foxn1nu/CrlNarl (BALB/c nude) mice, and C57BL/6J mice were sourced from BioLASCO (Taipei, Taiwan) and the National Laboratory Animal Center (Taipei, Taiwan), respectively. Experimental protocols were approved by the Institutional Animal Care and Use Committee of Chang Gung Memorial Hospital (CGMH).

### Cell lines and reagents

The intrahepatic cholangiocarcinoma (iCCA) cell lines HuCCT1 and KKU213 were obtained from the Japanese Collection of Research Bioresources (JCRB) Cell Bank (Osaka, Japan), whereas SSP-25 cells were sourced from the RIKEN BioResource Research Center (Tsukuba, Japan). Mouse iCCA ICKP cells were previously described 24. To establish GEM-resistant (GR) sublines, iCCA cells were grown in the media with an IC_90_ dose of GEM. After two to four months, the living cells were determined as gemcitabine-resistant (GR) sublines. SSP-25, HuCCT1, and their GR sublines were cultured in RPMI 1640 medium, whereas KKU213, KKU213-GR, ICKP, and HEK293 cells were maintained in high-glucose Dulbecco's modified Eagle's medium (DMEM). Both media were supplemented with 10% fetal bovine serum (FBS) and 1% penicillin-streptomycin. All cell lines were routinely tested for mycoplasma contamination and authenticated by short tandem repeat (STR) profiling, yielding negative and verified results. GEM (for the cell culture), dasatinib (for the cell culture), and cisplatin were purchased from Selleck Chemicals (Houston, TX, USA). Dasatinib (for mice) was purchased from AstraZeneca Plc. (Cambridge, England). GEM (for mice) was purchased from TTY Biopharm Co., Ltd (Taipei, Taiwan).

### Cell viability and combination index (*CI*)

Cell viability analyses were performed as previously described 24. Cells were seeded in 96-well plates at densities of 8 x 10^3^ cells per well (HuCCT1 and KKU213) and 5 x 10^3^ cells per well (SSP-25) and incubated overnight. Afterward, cells were treated with gradient concentrations of GEM or dasatinib for 72 h. Cell viability was evaluated using the WST-8 assay (Cell Counting Kit-8; Dojindo Molecular Technologies, Inc., Kumamoto, Japan) following the manufacturer's protocol. The half-maximal inhibitory concentration (IC_50_) values of GEM were determined using GraphPad Prism 11 software (GraphPad Software; San Diego, CA).

### cDNA microarray and RNA sequencing

Total RNA was extracted from cells with an A260/280 ratio greater than 1.9 and used for microarray analysis and RNA sequencing. cDNA microarray analysis was performed using the Affymetrix Human Genome U133 Plus 2.0 Array (Thermo Fisher Scientific, Inc., Waltham, MA, USA). Raw data were normalized against the parental SSP-25-GR cells. RNA sequencing results were prepared using a KAPA RNA HyperPrep Kit with RiboErase and were sequenced with Illumina NextSeq 550 to obtain 150 bp paired-end reads. Quantitative RT-PCR (RT-qPCR) analyses were performed as previously described 26. The primers for qPCR are listed in Table S3.

### Recombinant plasmids, virus production, and virus infection

The pCDH-GFP-VTCN1 or Src plasmid was generated by inserting full-length VTCN1 or Src into the pCDH-CMV-MCS-EF1α-copGFP vector (System Biosciences, LLC, Palo Alto, CA). The lentiviral packaging plasmids pCMV-ΔR8.91 and pMD.G, along with the pLKO.1-shRNA clones (Table S4), were obtained from the National RNAi Core Facility (Academia Sinica, Taipei, Taiwan). The processes of virus production and infection were performed as previously described 26.

### Immunoprecipitation (IP) and immunoblotting (IB)

For immunoprecipitation, cells were lysed in TGH buffer (50 mM HEPES [pH 7.4], 1% Triton X-100, 10% glycerol, 150 mM NaCl, 2 mM EDTA, and 2 mM EGTA) supplemented with a protease inhibitor cocktail (Roche, Mannheim, Germany). The lysates were incubated on ice for at least 60 min and then centrifuged at 12,000 g for 4 min at 4 °C. Immunoblotting was performed as previously described 26. The information on primary antibodies is listed in Table S5. Phospho-kinase array analysis was performed using the Human Phospho-Kinase Array Kit (ARY003C; R&D Systems, Minneapolis, MN, USA) according to the manufacturer's instructions. 800 μg whole-cell lysates were hybridized with the nitrocellulose membranes overnight and then with secondary antibodies at room temperature. Chemiluminescent images were captured, and relative signal intensities were analyzed using a UVP ChemStudio PLUS Touch imaging system running VisionWorks software (Analytik Jena AG, Jena, Germany).

### Immunofluorescence (IF) and proximity ligation assay (PLA)

Cells were fixed with 4% paraformaldehyde (PFA) in PBS for 30 min and subsequently blocked with 4% fetal bovine serum (FBS) in PBS for 2 h at room temperature. Cells were incubated with an anti-FLAG antibody (F3165; Sigma-Aldrich, St. Louis, MO, USA) in 4% FBS at 4 °C overnight, followed by a subsequent overnight incubation with secondary antibodies (Thermo Fisher Scientific, Inc., Waltham, MA, USA) at 4 °C. The proximity ligation assay was performed according to the manufacturer's protocols (Duolink® Proximity Ligation Assay kit, DUO92101; Sigma-Aldrich, St. Louis, MO). Briefly, cells were fixed, permeabilized, and blocked, followed by overnight incubation at 4 °C with a mixture of mouse and rabbit primary antibodies (Table S5) diluted in the blocking solution. The cells were incubated with PLA probes and underwent ligation and amplification. Coverslips were mounted using Fluoroshield Mounting Medium with DAPI (Abcam, Cambridge, UK) and subsequently visualized using a laser-scanning confocal microscope system (TCS SP8X; Leica Microsystems, Wetzlar, Germany). For PLA, the respective images in the figures were shown from a single Z-section.

### Conditioned media (CM) collection and sVTCN1 purification

To collect conditioned medium (CM), cells were cultured in dishes until they reached approximately 90% confluence, at which point the culture medium was replaced with serum-free medium. After 24 h of incubation, the CM was harvested. For Western blots, the proteins were precipitated with 20% trichloroacetic acid (TCA) and washed twice with cold acetone. The pellet fractions were collected and assayed by SDS-PAGE. For detecting VTCN1 concentrations, the CM was analyzed using a Human B7H4 DuoSet ELISA kit (DY6576-05; R&D Systems, Minneapolis, MN). To purify sVTCN1 from CM, HEK293 cells were transiently transfected to express FLAG-tagged VTCN1 for 24 h, followed by incubation in serum-free medium for an additional 24 h. The FLAG-tagged VTCN1 protein was then purified from the harvested CM using anti-FLAG beads (A36797; Thermo Fisher Scientific, Inc., Waltham, MA, USA) and subsequently eluted with Pierce™ 3x DYKDDDDK Peptide (A36805; Thermo Fisher Scientific). To evaluate the functional effects of sVTCN1, the purified protein was added to 1 mL of RPMI 1640 medium at a volume of either 10 μL (for experiments in Figure 5) or 20 μL (for experiments in Figure 6).

### Statistical analysis

Quantitative data are expressed as mean ± SD for *in vitro* experiments and mean ± SEM for *in vivo* studies. Differences between two groups were analyzed using a two-tailed independent Student’s *t*-test, whereas comparisons among multiple groups were evaluated by two-way ANOVA followed by Tukey's post-hoc test. Progression-free survival (PFS) and overall survival (OS) rates were estimated using the Kaplan-Meier method, and differences were assessed via the log-rank test. The log-rank test was also employed to evaluate the prognostic significance of various clinicopathological parameters in the univariate analysis. The Pearson correlation coefficient (r) was measured for a linear correlation between two variables. All statistical analyses were performed using GraphPad Prism software (version 11), with statistical significance defined as a two-tailed *P*-value < 0.05. The values of the combination index (*CI*) were calculated by CompuSyn software according to the user’s guide 27. The *CI* values were defined as follows: 0.1–0.3, strong synergism; 0.3–0.7, synergism 28.

## Results

### VTCN1 promotes GEM resistance (GR) in intrahepatic cholangiocarcinoma (iCCA) cells *in vitro*

**Figure 1:**
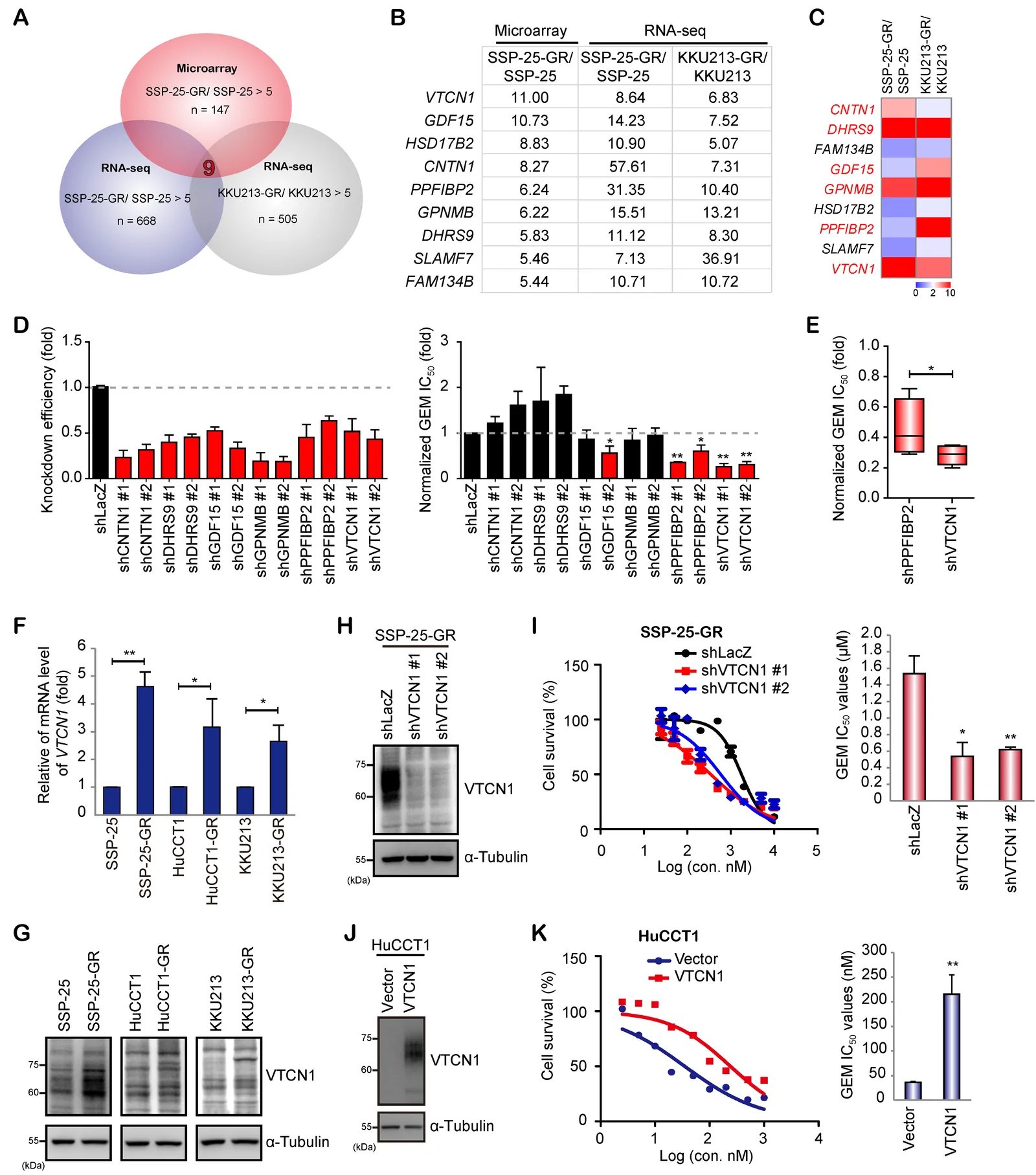
** VTCN1 triggers GEM resistance in iCCA. (A)** A schematic flowchart illustrating the identification of nine upregulated genes in iCCA gemcitabine-resistant (GR) sublines. **(B)** A table showing the fold changes of nine upregulated genes from GR sublines versus their parental iCCA cells. **(C)** A heatmap showing the relative mRNA levels detected by RT-qPCR in two GR pairs. Data are expressed as fold-changes relative to their respective parental counterparts. Red pixels: a 2-fold increase. **(D)** Left: The relative mRNA levels of six genes in HuCCT1-GR cells. Data are expressed as fold-changes relative to shLacZ. Red bars: downregulated expression. Right: The relative GEM IC50 values (mean ± SD, N = 3) in HuCCT1-GR cells receiving shRNA against six upregulated genes or a control sequence (shLacZ). The values are presented as the fold-change relative to cells receiving shRNA against a control sequence (shLacZ). **(E)** The relative GEM IC50 values (mean ± SD, N = 6) in HuCCT1-GR cells receiving two independent shRNAs against PPFIBP2 (shPPFIBP2), VTCN1 (shVTCN1), or a control sequence. The values are presented as the fold-change relative to cells receiving shRNA against a control sequence. **(F)** VTCN1 relative mRNA level was determined by RT-qPCR in three GR pairs. Data represent the mean ± SD (N = 3) of fold-changes normalized to parental cells. **(G)** WB displaying the protein levels of indicated targets in iCCA cells. **(H)** WB displaying the protein levels of VTCN1 and α-tubulin in the cells expressing shRNA targeting VTCN1 (shVTCN1) or a control sequence (shLacZ). **(I)** Cell viability curves (left) and GEM IC50 values (right; mean ± SD, N = 3) in SSP-25-GR cells expressing shRNA targeting VTCN1 (shVTCN1) or a control sequence (shLacZ). **(J)** WB displaying the protein levels of indicated targets in HuCCT1 cells overexpressing FLAG-tagged VTCN1 (VTCN1) or the empty control vector (Vector). **(K)** Cell viability curves (left) and GEM IC50 values (right; mean ± SD, N = 3) in HuCCT1 cells overexpressing FLAG-tagged VTCN1 (VTCN1) or the control vector (Vector). ** P < 0.005; *, P < 0.05 by Student’s t-test.

To identify molecular drivers of GR in iCCA, three GR iCCA sublines, SSP-25-GR 24, KKU213-GR, and HuCCT1-GR (Figure S1A-B) were established. The upregulated genes were analyzed by RNA-sequencing and cDNA microarray. Nine increased genes showed a 5-fold change in SSP-25-GR and KKU213-GR sublines (Figure 1A-B). The mRNA levels of nine increased genes were confirmed by RT-qPCR. Six genes (*CNTN1, DHRS9, GDF15, GPNMB, PPFIBP2,* and* VTCN1*) were upregulated in either one or both GR sublines (Figure 1C). Among the six candidate genes, shRNA-mediated knockdown of PPFIBP2 or VTCN1 significantly decreased the GEM IC_50_ values (Figure 1D). Compared to PPFIBP2, VTCN1 knockdown more markedly reduced GEM IC_50_ values (Figure 1E). VTCN1 (V-set domain containing T cell activation inhibitor 1, B7H4) belongs to the B7 coinhibitory protein family. To confirm the increased expression, the mRNA and protein expression levels of VTCN1 were analyzed in three iCCA GR pairs. The increased mRNA and protein levels were detected in three GR sublines (Figure 1F-G). According to the data, VTCN1 expression was hypothesized to mediate GEM resistance in iCCA. The knockdown of VTCN1 by shRNAs decreased GEM IC_50_ values in SSP-25-GR and HuCCT1-GR cells (Figure 1H-I; Figure S1C-D). On the contrary, VTCN1 overexpression in parental GEM-sensitive iCCA cells increased GEM IC_50_ values (Figure 1J-K; Figure S1E-F). Those results suggest that reduced VTCN1 enhances GEM sensitivity in iCCA, and vice versa.

### High VTCN1 expression predicts GEM non-response and soluble VTCN1 (sVTCN1) correlates with tumor burden

**Figure 2:**
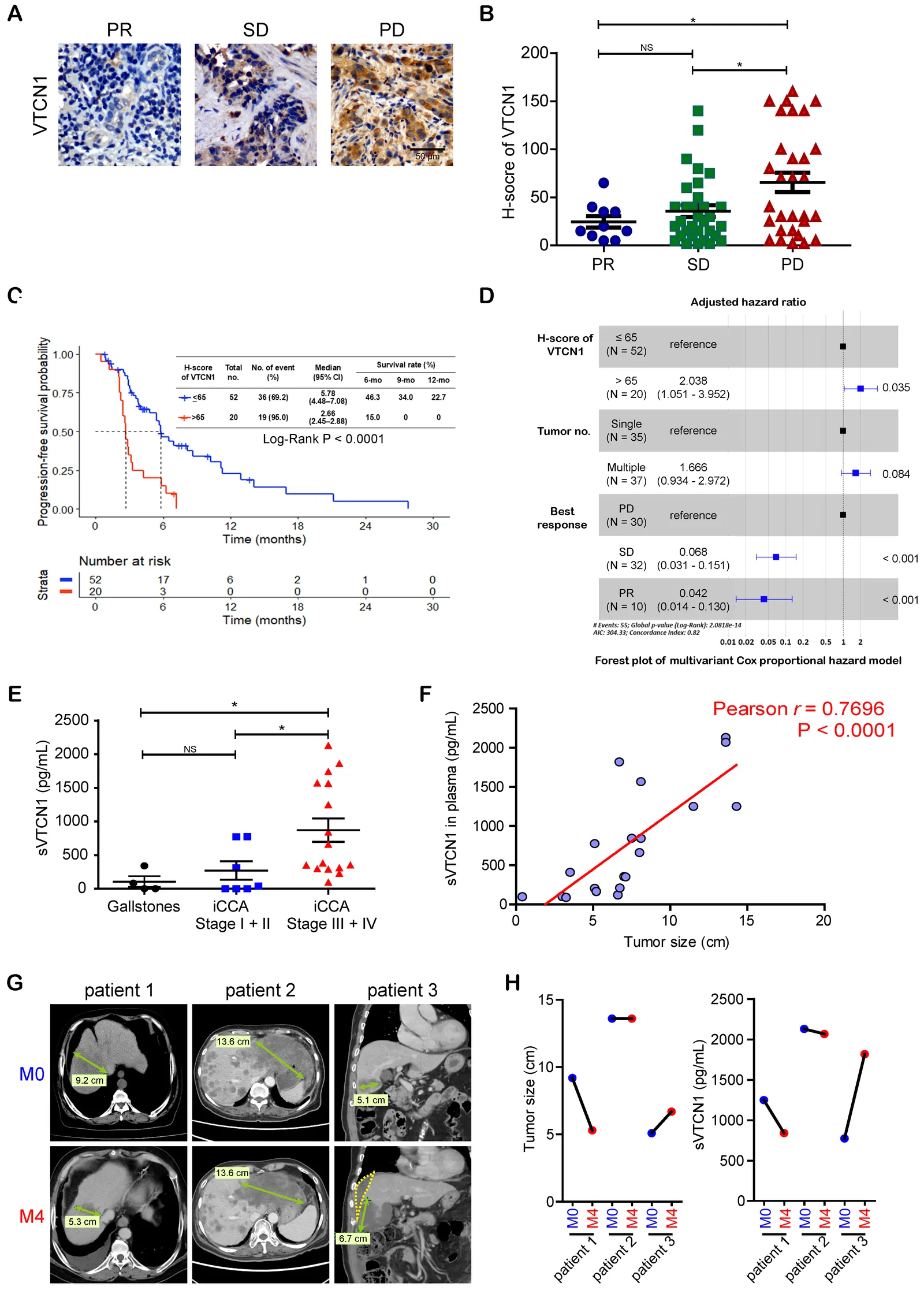
** VTCN1 predicts GEM response and sVTCN1 correlates with tumor burden. (A)** Representative micrographs of VTCN1 immunohistochemical (IHC) staining in GEM-treated iCCA patients. Patients are categorized by clinical response: PR (n = 10, partial response), SD (n = 32, stable disease), and PD (n = 30, progressive disease). Scale bar = 50 μm. **(B)** Distribution of VTCN1 H-scores in iCCA patients with varying GEM responses. **(C)** Kaplan-Meier plot of the progression-free survival (PFS) of iCCA patients with low VTCN1 expression (VTCN1 ≤ 65, n = 52, blue) or high VTCN1 expression (VTCN1 > 65, n = 20, red). The optimal cut-off point for the H-score of the VTCN1 variable was determined using maximally selected rank statistics from the maxstat R package. Median PFS: 5.78 months vs. 2.66 months. The P value was determined by the log-rank test. **(D)** Forest plot based on hazard ratios from Cox regression for all subgroups after adjustment for centers. Squares represent hazard ratios. Data represent 95% confidence intervals. P values were calculated via the log-rank test. **(E)** The levels of soluble VTCN1 (sVTCN1) in blood plasma samples from the patients with gallstones (n = 4) or iCCA (Stage I + II, n = 7; Stage III + IV, n = 16). **(F)** Correlation analysis between primary tumor sizes in computed tomography (CT) images and plasma soluble VTCN1 (sVTCN1) levels from 20 iCCA patients. The Pearson correlation coefficient (r) and P values are indicated in the panel. **(G)** The CT images are obtained from three iCCA patients at the indicated time points (M, month). The green double-headed arrows indicate the measured dimensions of iCCA tumors. The yellow dotted line in patient 3 indicates the new tumor burden after 4 months (M4). **(H)** The iCCA tumor sizes and the levels of sVTCN1 in blood plasma samples of three iCCA patients at the indicated time points. M0, month 0; M4, month 4 *, P < 0.05 by Student’s t-tests. NS, non-significant.

We also investigated the previous results in iCCA patients. In patient specimens, VTCN1 expression levels were significantly lower in GEM-treated patients achieving a partial response (PR) or stable disease (SD) compared to those with progressive disease (PD; Figure 2A-B). iCCA patients with high VTCN1 expression exhibited significantly shorter progression-free survival (PFS) than those with low expression, whereas no significant association was observed with overall survival (OS; Figure 2C; Figure S2A). Multivariate Cox regression analysis identified VTCN1 expression as an independent prognostic factor for PFS after adjusting for tumor number and treatment response (HR = 2.038, 95% CI = 1.051-3.952, P = 0.035; Figure 2D).

Consistently, VTCN1, tumor numbers, and poor response showed significant association with PFS in univariate analysis (Table 1). The soluble form of VTCN1 (sVTCN1) in the peripheral blood is detected in patients with autoimmune diseases 29. In the iCCA cell culture system, sVTCN1 protein was found in the CM, and increased sVTCN1 was detected in VTCN1 overexpressing cells and GR sublines (Figure S2B-C). sVTCN1 levels in the blood plasma samples were positively correlated with VTCN1 protein expression in the primary iCCA tumors (Figure S2D). sVTCN1 levels in the blood plasma samples from iCCA patients with late-stage iCCA were higher than those with early-stage iCCA or gallstones (Figure 2E), and the levels were positively associated with primary tumor sizes (Figure 2F). The decreased primary tumor was accompanied by the downregulation of sVTCN1 level, and the increased tumor displayed the upregulated sVTCN1 level (Figure 2G-H). Collectively, high VTCN1 expression was detected in GEM non-responders, and sVTCN1 levels were positively associated with tumor burden.

### Src activation mediates VTCN1-driven GR in iCCA

**Figure 3:**
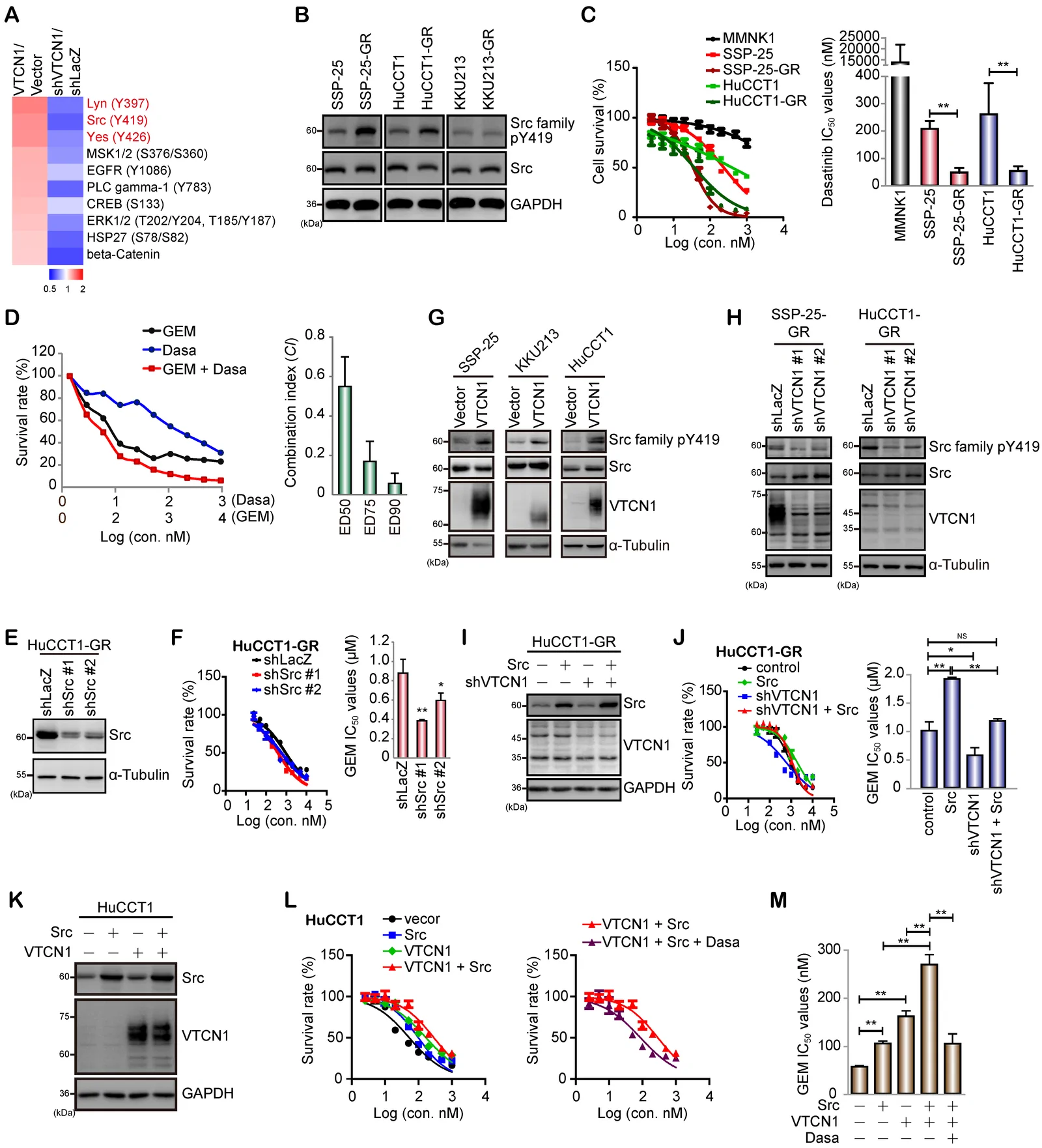
** VTCN1 reduces GEM sensitivity via Src activation in iCCA *in vitro*. (A)** A heatmap showing the top 10 differentially phosphorylated proteins in modified iCCA cells relative to their respective controls. The analysis contrasts VTCN1-overexpressed cells (HuCCT1-VTCN1) with the empty vector control (HuCCT1-Vector), and VTCN1-depleted cells (SSP-25-GR/shVTCN1) with a control sequence (SSP-25-GR/shLacZ). Red and blue pixels represent upregulated and downregulated phosphorylation levels. **(B)** WB displaying the protein levels of indicated targets in various iCCA cell lines. **(C)** Left: Cell viability curves of iCCA and MMNK1 cholangiocytes following a 72 h treatment with gradient concentrations of dasatinib. Right: Corresponding IC50 values of dasatinib in the specified cell lines, presented as mean ± SD (N = 3). **(D)** Left: Cell viability curves of HuCCT1 cells following a 72 h treatment with gradient concentrations of dasatinib (Dasa) and GEM for 72 h. Right: Combination index (CI) values (mean ± SD, N= 3) for the combined treatment in HuCCT1 cells. ED, effective dose. **(E)** WB displaying the protein levels of indicated targets in the cells expressing shRNA targeting Src (shSrc) or a control sequence (shLacZ). **(F)** Cell viability curves (left) and GEM IC50 values (right; mean ± SD, N = 3) in HuCCT1-GR cells receiving shRNA against Src (shSrc) or a control sequence (shLacZ). **(G)** WB displaying the protein levels of indicated targets in three iCCA cell lines overexpressing FLAG-tagged VTCN1 (VTCN1) or the empty control vector (Vector). **(H)** WB displaying the protein levels of indicated targets in those cells expressing shRNA targeting VTCN1 (shVTCN1) or a control sequence (shLacZ). **(I)** WB displaying the protein levels of indicated targets in VTCN1-depleted (shVTCN1, +) HuCCT1-GR cells receiving Src overexpression (Src, +) or the empty control vector (Src, -). **(J)** Cell viability curves (left) and GEM IC50 values (right; mean ± SD, N = 3) in VTCN1-depleted (shVTCN1) HuCCT1-GR cells receiving Src overexpression or a control vector. **(K)** WB displaying the protein levels of indicated targets in VTCN1-overexpressed (VTCN1, +) HuCCT1 cells receiving Src overexpression (Src, +) or the empty control vector (Src, -). **(L)** Cell viability curves in VTCN1-overexpressed HuCCT1 cells receiving Src overexpression or a control vector (left) and in the presence of DMSO or 100 nM dasatinib (right, Dasa) for 72 h in HuCCT1-overexpressed VTCN1 and Src cells. **(M)** GEM IC50 values (right; mean ± SD, N = 3) in VTCN1-overexpressed HuCCT1 cells receiving Src overexpression (Src, +) or a control vector (Src, -) and in the presence of DMSO (Dasa, -) or 100 nM dasatinib (Dasa, +) for 72 h in HuCCT1-overexpressed VTCN1 and Src cells. ** P < 0.005; *, P < 0.05 by Student’s t-test. NS, non-significant.

Next, we investigated how VTCN1 mediates GR in iCCA. The phosphorylation of 37 kinases and the expression of two related proteins were analyzed using a commercial phospho-kinase array kit. The phosphorylation levels of Src family proteins (Lyn, Src, and Yes) were markedly upregulated in HuCCT1 cells with VTCN1 overexpression and were downregulated in SSP-25-GR cells with VTCN1 depletion (Figure 3A; Figure S3A; Table S2). The upregulated Src family protein phosphorylation was determined in GR cells compared to their parental cells (Figure 3B). Although VTCN1 was reported to affect the JAK-STAT3 axis 30 and AKT phosphorylation 31, the phosphorylation levels of those proteins were not mediated by VTCN1 overexpression in iCCA cells (Figure S3B). To elucidate the role of Src activity in mediating GEM resistance, Src family kinases were pharmacologically inhibited using dasatinib. Dasatinib induced more iCCA cell death compared with the immortalized cholangiocytes, MMNK1 cells. Notably, GR cells were more sensitive to dasatinib than their parental cells (Figure 3C). Dasatinib treatment reduced GEM IC_50_ values in HuCCT1-GR cells (Figure S3C). Concomitant treatment with dasatinib and GEM produced synergy in both parental HuCCT1 (*CI* = 0.1 - 0.3 in ED75 and ED90; Figure 3D) and resistant HuCCT1-GR cells (*CI* = 0.3 - 0.7; Figure S3D).

Among the Src family proteins, Fyn, Lyn, Src, and Yes can be detected in iCCA cells, but Lck expression was mainly detected in Jurkat (T cell leukemia) cells and was hard to detect in iCCA cells (Figure S3E)*.* Knockdown of Src by shRNAs reduced GEM IC_50_ values in HuCCT1-GR and SSP-25-GR sublines (Figure 3E-F; Figure S3F-G). The GEM IC_50_ values were not markedly reduced in Fyn, Lyn, or Yes knockdown HuCCT-GR cells (Figure S3H-I), suggesting that Fyn, Lyn, or Yes did not affect GEM resistance in iCCA cells. To further validate these findings from the phospho-kinase array, the effect of VTCN1 expression on the phosphorylation of the Src family was validated in iCCA cells. VTCN1 overexpression enhanced the phosphorylation of Src family proteins in three iCCA cells (Figure 3G), and the knockdown of VTCN1 reduced the phosphorylation in GR sublines (Figure 3H). To evaluate whether Src activity contributes to VTCN1-mediated GEM resistance, Src was overexpressed in VTCN1-depleted HuCCT1-GR cells. Src overexpression increased GEM IC_50_ values and could compensate for the decreased values caused by VTCN1 depletion (Figure 3I-J), suggesting that Src is sufficient for VTCN1-mediated GEM resistance. Moreover, overexpression of VTCN1 increased GEM IC_50_ values, and Src overexpression enhanced these values. The increased effects were suppressed by Src family inhibitor dasatinib treatment in SSP-25 and HuCCT1 cells (Figure 3K-M; Figure S3J-L*).* Taken together, those results suggest that VTCN1 promotes GEM resistance by promoting Src phosphorylation in iCCA.

### The interaction of VTCN1 and integrin β1 promotes integrin-dependent Src activation

The topological domains of VTCN1 contain an extracellular domain (a.a. 25-259), a trans-membrane helical domain (a.a. 260-280), and a cytoplasmic domain (a.a. 281-282) 2. Since there are few amino acids to associate with cytoplasmic effectors, we hypothesized that VTCN1 mediates Src phosphory-lation via interacting with other membrane proteins. Src family proteins are activated and phosphorylated by two major signals: growth factor-dependent signals and integrin-dependent signals 32. In integrin-dependent signals, integrins promote Src activation via focal adhesion kinase (FAK) 33. In the phospho-kinase array (Figure 3A), EGFR phosphorylation was upregulated in VTCN1-overexpressed cells and downregulated in VTCN1-depleted cells.

**Figure 4:**
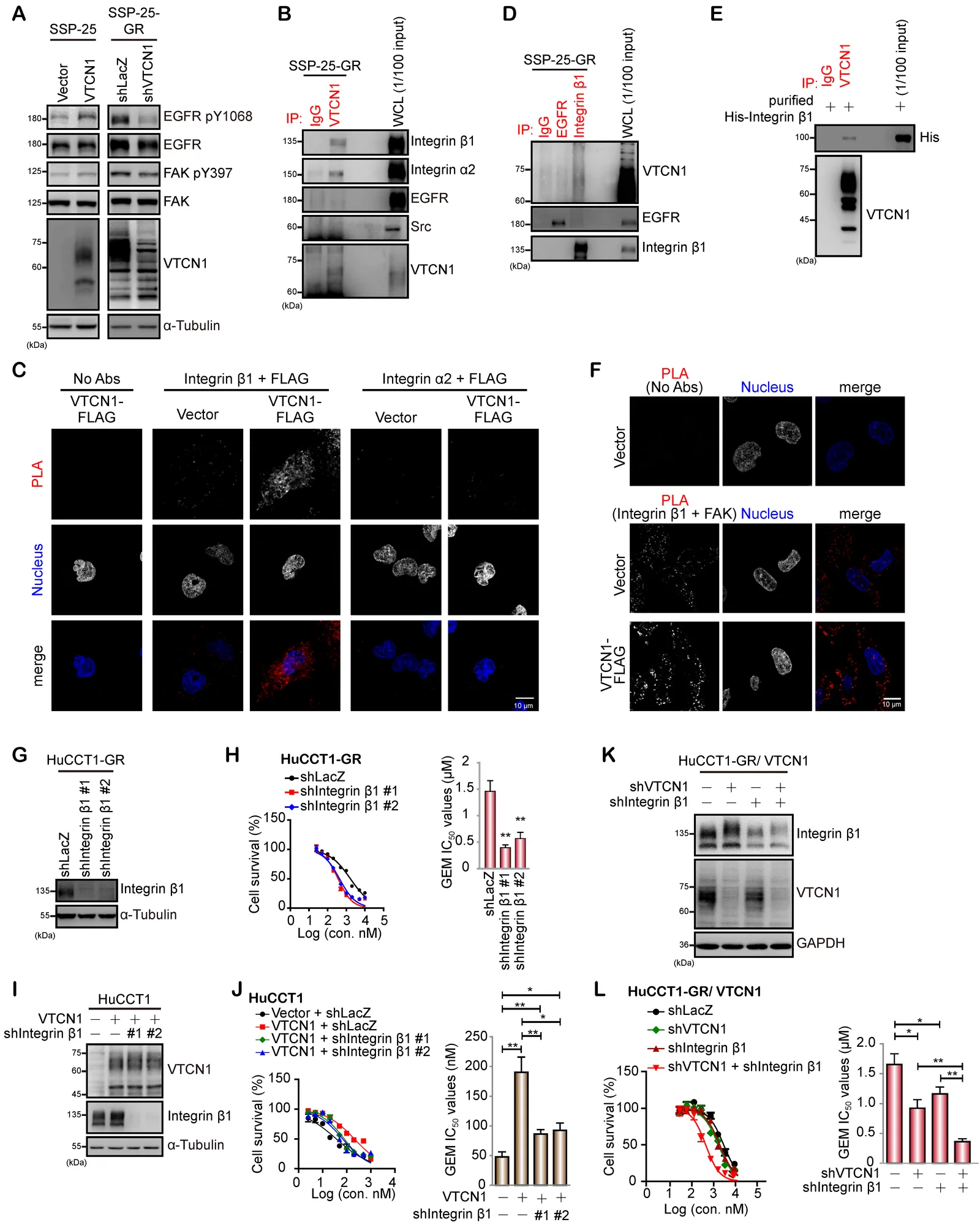
** VTCN1 interacts with integrin β1 to activate the FAK-Src axis. (A)** Left: WB displaying the protein levels of the indicated targets in the cells overexpressing FLAG-tagged VTCN1 (VTCN1) or the empty control vector (Vector). Right: WB displaying the protein levels of the indicated targets in the cells expressing shRNA targeting VTCN1 (shVTCN1) or a control sequence (shLacZ). **(B)** Immunoprecipitation (IP)-WB showing the interaction between VTCN1 and integrins in SSP-25-GR cells. The immunocomplexes, immunoprecipitated (IP) using an anti-VTCN1 antibody or a control IgG, and whole-cell lysates (WCL) were analyzed with the indicated antibodies. **(C)** A duolink proximity ligation assay (PLA) for showing the interactions between FLAG-tagged VTCN1 (VTCN1-FLAG) and integrin β1 or integrin α2 *in situ*. SSP-25 cells expressing FLAG-tagged VTCN1 or a control vector (Vector) were grown on coverslips overnight. A duolink assay was performed and DNA was stained with DAPI (blue). The red spots in PLA indicated integrin β1 and FLAG-tagged VTCN1 interaction. **(D)** IP-WB blot showing the interaction between VTCN1 and EGFR or integrin β1 in SSP-25-GR cells. The immunocomplexes, immunoprecipitated (IP) using an anti-EGFR antibody, anti-integrin β1 antibody, or a control IgG, and whole-cell lysates (WCL) were analyzed with the indicated antibodies. **(E)** A pull-down assay for demonstrating the direct interaction of integrin β1 and VTCN1. FLAG-tagged VTCN1 was immunoprecipitated (IP) from SSP-25 cells by VTCN1 antibody or a control IgG. The immunocomplexes were incubated with purified His-integrin β1 (a.a. 21-728, ab219474) and then analyzed by immunoblots (IB) with the indicated proteins. **(F)** A duolink proximity ligation assay (PLA) for showing the effect of VTCN1 overexpression on integrin β1 and FAK interaction *in situ*. SSP-25 cells expressing FLAG-tagged VTCN1 or a control vector (Vector) were grown on coverslips overnight. A duolink assay was performed and DNA was stained with DAPI (blue). The red spots in PLA indicated integrin β1 and FAK interaction. **(G)** WB displaying the protein levels of indicated targets in HuCCT1-GR cells expressing shRNA targeting integrin β1 (shIntegrin β1) or a control sequence (shLacZ). **(H)** Cell viability curves (left) and GEM IC50 values (right; mean ± SD, N = 3) in HuCCT1-GR cells expressing shRNA targeting integrin β1 (shIntegrin β1) or a control sequence (shLacZ). **(I)** WB displaying the protein levels of indicated targets in VTCN1-overexpressed HuCCT1 cells receiving shRNA targeting integrin β1 (shIntegrin β1) or the empty control sequence (shIntegrin β1, -). **(J)** Cell viability curves (left) and GEM IC50 values (right; mean ± SD, N = 3) in VTCN1-overexpressed HuCCT1 cells receiving shRNA targeting integrin β1 (shIntegrin β1) or a control sequence (shLacZ). **(K)** WB showing the protein levels of indicated targets in VTCN1-overexpressed HuCCT1-GR (HuCCT1-GR/ VTCN1) cells receiving shRNA targeting VTCN1 (shVTCN1), integrin β1 (shIntegrin β1), or the empty control sequence (shLacZ). **(L)** Cell viability curves (left) and GEM IC50 values (right; mean ± SD, N = 3) in VTCN1-overexpressed HuCCT1-GR (HuCCT1-GR/ VTCN1) cells receiving shRNA targeting VTCN1 (shVTCN1), integrin β1 (shIntegrin β1), or the control sequence (shLacZ). ** P < 0.005; *, P < 0.05 by Student’s t-test.

Indeed, EGFR Tyr-1068 phosphorylation and FAK Tyr-397 phosphorylation were improved upon VTCN1 overexpression and impaired upon VTCN1 knockdown (Figure 4A). Among integrin subunits, integrin α2 and integrin β1 have been demonstrated to promote GEM resistance 34, 35. Thus, we confirmed the role of integrin α2 and integrin β1 in VTCN1-mediated Src phosphorylation. VTCN1 was precipitated by VTCN1 antibody. Integrin β1 and integrin α2, but not EGFR and Src, were detected in the immuno-complex, suggesting that VTCN1 can associate with integrin β1 and integrin α2, but not EGFR and Src (Figure 4B). To confirm whether VTCN1 directly interacts with integrin α2 or integrin β1, an *in situ* proximity ligation assay (PLA) was performed. PLA revealed that VTCN1-FLAG formed a complex with integrin β1, but not integrin α2 and EGFR, in iCCA cells (Figure 4C; Figure S4A), and VTCN1-integrin β1 interaction was also detected in the integrin β1-precipitated complex (Figure 4D). Moreover, VTCN1-FLAG can directly interact with purified His-tagged integrin β1 (Figure 4E). In integrin signaling, integrins recruit and promote FAK phosphorylation, disrupting Src auto-inhibition to induce Src auto-phosphorylation 36. To clarify the effect of VTCN1 on integrin β1-mediated signals, the association of integrin β1 and FAK was analyzed. Increased VTCN1 expression enhanced integrin β1 and FAK interaction at cell membranes (Figure 4F; Figure S4B-C).

In terms of functional relevance, knockdown of integrin β1 successfully reduced GEM IC₅₀ values in HuCCT1-GR cells (Figure 4G-H). VTCN1 overexpression upregulated GEM IC_50_ values, and the increases caused by VTCN1 overexpression were partially suppressed by integrin β1 knockdown, suggesting that integrin β1 partially contributes to VTCN1-mediated GEM resistance (Figure 4I-J). To detect the synergistic effects of VTCN1 and integrin β1, VTCN1 and integrin β1 were depleted by shRNA in GR cells. VTCN1 or integrin β1 knockdown reduced GEM IC_50_ values. The dual knockdown of VTCN1 and integrin β1 caused the beneficial effect in decreasing GEM IC_50_ values compared to VTCN1 knockdown or integrin β1 knockdown alone (Figure 4K-L). The data suggest that VTCN1 is directly associated with integrin β1, resulting in Src phosphorylation and enhancing GEM resistance.

### sVTCN1 induces a positive feedback loop to bind EGFR, resulting in the activation of the EGFR-Src axis

**Figure 5:**
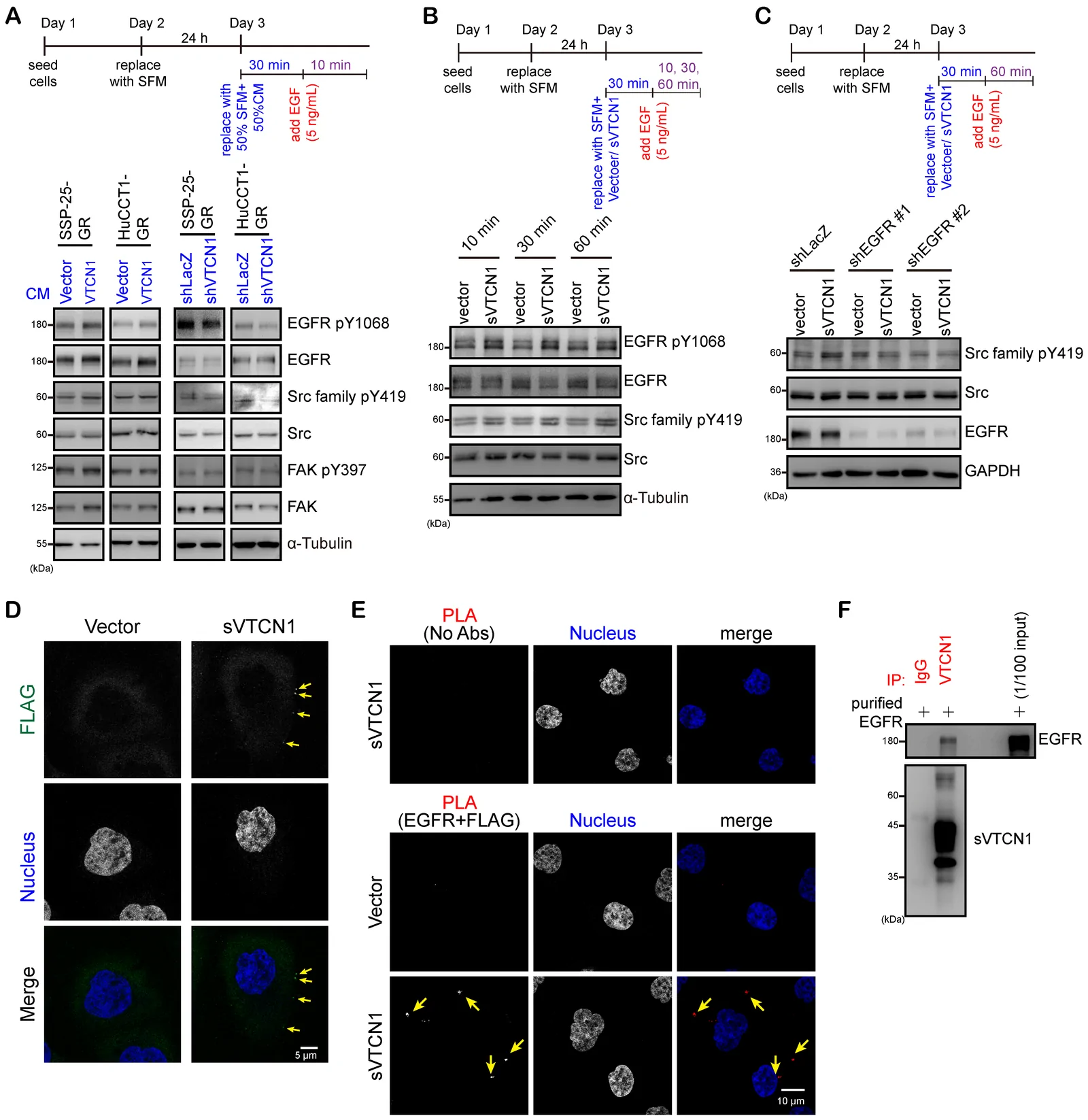
** Soluble VTCN1 (sVTCN1) interacts with EGFR to enhance Src activation, resulting in GR in iCCA. (A)** Upper: A schema for the experimental design for the lower WB. Lower: WB showing the protein levels of indicated targets in the conditioned media (CM)-treated SSP-25-GR cells or HuCCT1-GR cells. Lower left: the CM was collected from HEK293 cells overexpressing FLAG-tagged VTCN1 (VTCN1, blue words) or a control vector (Vector, blue words). Lower right: the CM were collected from SSP-25-GR cells receiving shRNA against VTCN1 (shVTCN1, blue words) or a control sequence (shLacZ, blue words). SSP-25-GR cells or HuCCT1-GR cells were grown in serum-free media (SFM) for 24 h, treated with the CM for 30 min, and then treated with the combination of the CM and EGF (5 ng/mL) for 10 min. **(B)** Upper: A schema for the experimental design for the lower WB. Lower: WB showing the protein levels of indicated targets. HEK293 cells overexpressing VTCN1 or a control vector, and soluble VTCN1 (sVTCN1) was purified from the conditioned media (CM). SSP-25-GR cells were grown in serum-free media (SFM) for 24 h, treated with sVTCN1 (10 μL sVTCN1 per mL of SFM, Figure S5D) or control proteins (Vector) for 60 min, and then EGF (5 ng/mL) for another 10 min, 30 min, or 60 min. **(C)** Upper: A schema for the experimental design for the lower WB. Lower: WB showing the protein levels of indicated targets. HEK293 cells overexpressing VTCN1 or a control vector, and soluble VTCN1 (sVTCN1) was purified from the CM. SSP-25-GR cells receiving shRNA against EGFR (shEGFR #1 and #2) or a control sequence (shLacZ) were grown in serum-free media (SFM) for 24 h, treated with sVTCN1 (10 μL sVTCN1 per mL of SFM, Figure S5D) or control proteins (Vector) for 60 min, and then added EGF (5 ng/mL) for another 60 min. **(D)** SSP-25-GR cells were grown on coverslips overnight and then treated with the CM from HEK293 cells overexpressing FLAG-tagged VTCN1 (VTCN1-FLAG) or a control vector (Vector) for another 24 h. The cells were fixed and stained with FLAG (green) and DNA with DAPI (blue). Yellow arrows, FLAG-positive signals. Scale bar = 5 μm. **(E)** A duolink proximity ligation assay (PLA) for showing the interactions between sVTCN1 and EGFR *in situ*. SSP-25-GR cells were grown on coverslips overnight and then treated with the CM from HEK293 cells overexpressing FLAG-tagged VTCN1 (VTCN1-FLAG) or a control vector (Vector) for another 24 h. A Duolink assay was performed, and DNA was stained with DAPI (blue). The red spots (yellow arrows) in PLA indicated the EGFR and sVTCN1-FLAG interaction. Scale bar = 10 μm. **(F)** A pull-down assay for demonstrating the direct interaction of EGFR and sVTCN1. sVTCN1 were immunoprecipitated (IP) from VTCN1 overexpressing SSP-25 cell-derived CM by VTCN1 antibody or control IgG. The immunocomplexes were incubated with purified FLAG-EGFR WT and then analyzed by immunoblots with the indicated proteins.

VTCN1 overexpression increased Src Tyr-419 phosphorylation and GEM IC_50_ values, and the increases caused by VTCN1 overexpression were partially suppressed by integrin β1 knockdown (Figure 4I-J; S5A), indicating that additional signaling pathways contribute to VTCN1-mediated Src activation. VTCN1 also induced EGFR Tyr-1068 phosphorylation, but the increase was not affected by integrin β1 knockdown (Figure S5A). EGFR Tyr-1068 phosphorylation was mediated by VTCN1 expression (Figure 3A and 4A), and VTCN1 expression was negatively correlated with the sensitivity of ErbB family-related TKIs (Figure S5B). However, the interaction of VTCN1 and EGFR was not detected in iCCA cells (Figure 4B). sVTCN1 was detected in the blood plasma from iCCA patients and CM from iCCA cells (Figure 2). Thus, we assumed that EGFR phosphorylation may be affected by sVTCN1. Under the low-dose EGF (5 ng/mL) stimulation, the treatment of CM from VTCN1 overexpressing cells enhanced the phosphorylation of EGFR Tyr-1068, but not FAK phosphorylation compared with the cells treated with the treatment of CM from control cells. Conversely, the treatment of CM from VTCN1-depleted cells reduced EGFR phosphorylation status compared with the treatment of CM from control shRNA (Figure 5A). To further confirm sVTCN1-promoted EGFR phosphorylation, sVTCN1 was purified from CM by FLAG beads (Figure S5C-D). Under low-dose EGF (5 ng/mL) stimulation, sVTCN1 (10 μL; Figure S5D) addition increased EGFR and Src phosphorylation compared with control treatment in SSP-25-GR cells (Figure 5B), and the increased Src phosphorylation was suppressed by EGFR knockdown (Figure 5C). To clarify whether sVTCN1 affects EGFR phosphorylation by directly binding to EGFR, the association of EGFR and sVTCN1 was analyzed. FLAG-tagged sVTCN1 associated with iCCA cells (Figure 5D) and sVTCN1 bound to EGFR (Figure 5E). The pull-down experiments demonstrated that sVTCN1 directly interacted with EGFR (Figure 5F). These findings establish sVTCN1-promoted Src phosphorylation via direct interaction with EGFR.

### sVTCN1 promotes GEM insensitivity via a Src-dependent manner in iCCA

**Figure 6:**
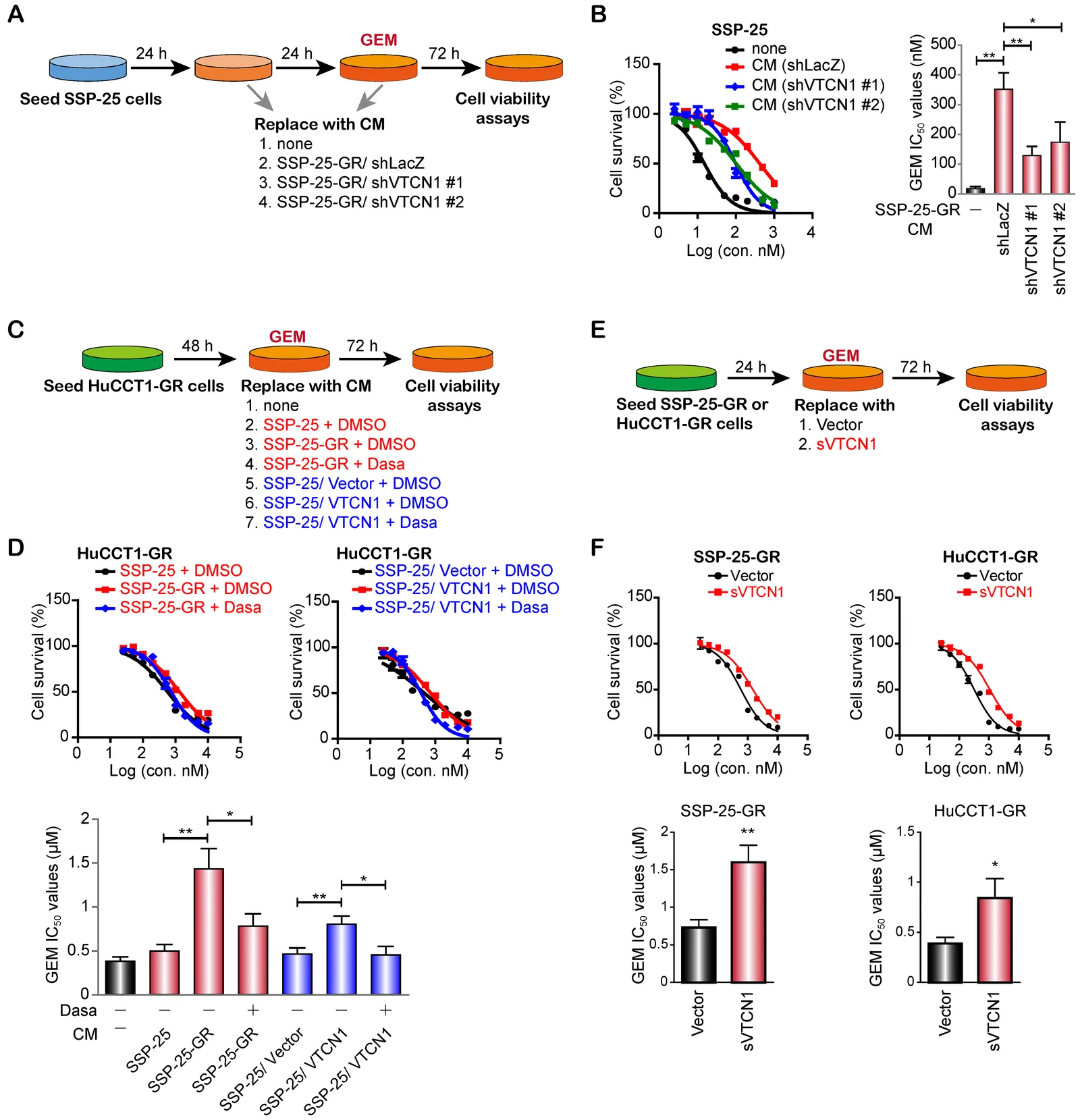
** sVTCN1 and CM-mediated GEM insensitivity requires Src activation. (A)** A schematic diagram illustrating the experimental design for the cell viability assay presented in panel B. The conditioned media (CM) were collected from SSP-25-GR cells receiving shRNA against VTCN1 (shVTCN1) or a control sequence (shLacZ). SSP-25 cells were grown for 24 h, treated with or without (none) the CM for 24 h, and then with various concentrations of GEM alone (none) or the combination of the CM and various concentrations of GEM for another 72 h. **(B)** Left: Cell viability curves of SSP-25 cells cultured in the presence or absence (none) of CM across a range of GEM concentrations. Right: Corresponding GEM IC50 values (mean ± SD, N = 3).** (C)** A schematic diagram illustrating the experimental design for the cell viability assay presented in panel D. The CM were collected from SSP-25 cells, SSP-25-GR cells, or SSP-25 cells overexpressing FLAG-tagged VTCN1 (VTCN1) or a control vector (Vector). HuCCT1-GR cells were seeded for 48 h and then treated for 72 h with gradient concentrations of GEM alone, or in combination with CM and either 100 nM dasatinib (Dasa) or an equal volume of DMSO as the vehicle control. **(D)** Upper: Cell viability curves of HuCCT1-GR cells treated with the combination of the CM and 100 nM dasatinib (Dasa) or DMSO with gradient concentrations of GEM. Lower: The GEM IC50 values (mean ± SD, N = 3) in HuCCT1-GR cells treated with or without (-) the combination of the CM and 100 nM dasatinib (Dasa, +) or DMSO (-). **(E)** A schematic diagram illustrating the experimental design for the cell viability assay presented in panel F. sVTCN1 was purified from CM from HEK293 cells overexpressing FLAG-tagged VTCN1 (sVTCN1) or a control vector (Vector). SSP-25-GR or HuCCT1-GR cells were grown for 24 h and then treated with various concentrations of GEM and sVTCN1 (20 μL sVTCN1 per mL of media, Figure S5D) or a control (Vector) for another 72 h. **(F)** Upper: Cell viability curves of SSP-25-GR and HuCCT1-GR cells treated with purified sVTCN1 or a control vector across various GEM concentrations. Lower: Corresponding GEM IC50 values (mean ± SD, N = 3). ** P < 0.005; *, P < 0.05 by Student’s t-test.

To examine whether sVTCN1 affects GEM sensitivity, iCCA cells were treated with iCCA-derived CM, and GEM IC_50_ values were determined. The treatment of GR cells-derived CM increased GEM IC_50_ values in SSP-25 cells, and the increased values were reduced by the treatment of VTCN1-depleted GR cells-derived CM (Figure 6A-B). To further investigate the effect of dasatinib on the CM-mediated GEM sensitivity, a combined treatment of CM and dasatinib in HuCCT1-GR and SSP-25 cells was performed. In both cells, higher GEM IC_50_ values were detected in SSP-25-GR-derived CM-treated cells compared with those in SSP-25-derived CM-treated cells. Dasatinib impaired the upregulated GEM IC_50_ values induced by SSP-25-GR-derived CM-treatment (Figure 6C-D; Figure S6A-B). Consistently, increased GEM IC_50_ values were determined in HuCCT1-GR cells treated with the CM from VTCN1 overexpressing SSP-25 cells, and dasatinib treatment repressed the increased values (Figure 6C-D). Furthermore, the addition of purified sVTCN1 (20 μL; Figure S5D) increased GEM IC_50_ values in two GR sublines (Figure 6E-F). These results demonstrate that the treatment of sVTCN1 or CCA-derived CM increased GEM insensitivity, and the increase was repressed by dasatinib or VTCN1 knockdown.

### VTCN1-Src axis enhances NT5C3 expression to reduce GEM sensitivity in iCCA cells

**Figure 7:**
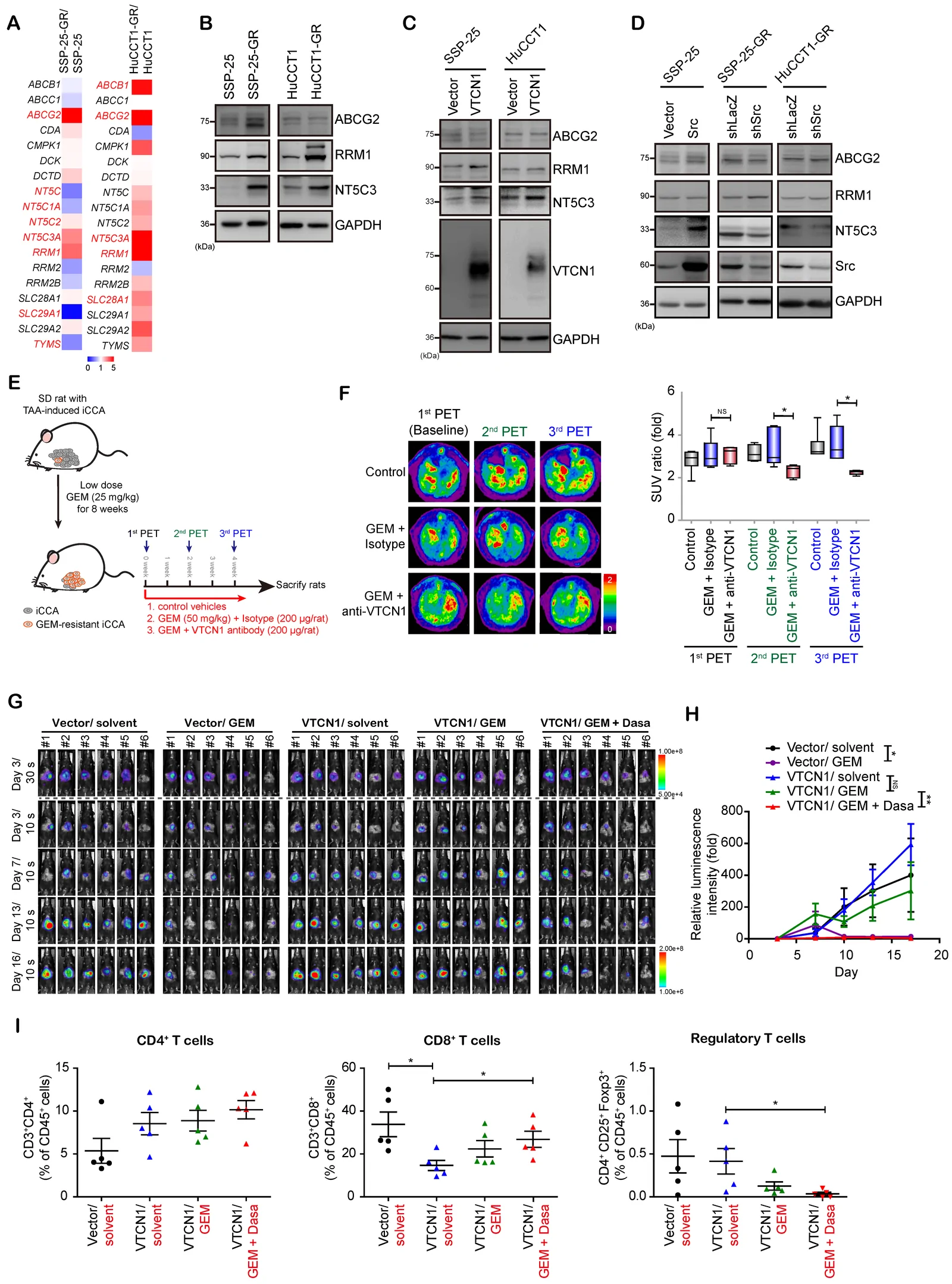
** NT5C3 is upregulated by the VTCN1-Src axis, and the axis is validated *in vivo*. (A)** A heatmap illustrating the mRNA expression levels of GEM metabolism-related genes in two GR sublines (SSP-25-GR and HuCCT1-GR) compared with those of their respective parental cells (SSP-25 and HuCCT1). Red pixels, upregulated expression; blue pixels, downregulated expression. Red words, significant gene expression changes. **(B)** WB showing the protein levels of indicated targets in parental and GR iCCA cell lines. **(C)** WB displaying the levels of indicated targets in the cells overexpressing VTCN1 or a control vector (Vector). **(D)** WB displaying the levels of indicated targets in SSP-25 overexpressing VTCN1 or a control vector (Vector), or SSP-25-GR or HuCCT1-GR cells receiving shRNA against Src (shSrc), or a control sequence (shLacZ). **(E)** An experimental schema in a spontaneous iCCA rat model. Sprague-Dawley rats received thioacetamide (TAA, 300 mg/L) in drinking water for 30-35 weeks to induce iCCA. Two-phase design: (1) Low-dose GEM (25 mg/kg, 8 weeks) to establish resistance; (2) Full-dose GEM (50 mg/kg) ± anti-VTCN1 antibody (4 weeks) to test therapeutic intervention in resistant disease. After 8 weeks, the rats were randomized into three groups: (1) control vehicle (n = 6), (2) GEM (50 mg/kg, weekly) + isotype control antibody (200 μg, three times per week, n = 5), or (3) GEM + anti-VTCN1 antibody (n = 5) for 4 weeks. Tumor burden was assessed by ¹⁸F-FDG PET/CT at baseline, 2 weeks, and 4 weeks. **(F)** Left: Representative PET images of TAA-induced iCCA in SD rats. Scans were performed at baseline (1st PET, before treatment, Baseline), and subsequently after 2 weeks (2nd PET) and 4 weeks (3rd PET) of treatment. Right: The values of the tumor-to-liver (T/L) ratio of SUV before treatment (1st PET; Baseline) and after 2 weeks (2nd PET) and 4 weeks (3rd PET). N ≥ 5 for each group. Box-and-whisker plots show the distribution of the data: maximum, upper quartile, median, lower quartile, and sample minimum. *, P < 0.05 by Student's t-test. NS, non-significant. **(G)** ICKP cells stably overexpressing VTCN1 or an empty control vector were orthotopically injected into the left lobe of the liver in C57BL/6J mice. GEM (25 mg/kg) was administered by intraperitoneal injection twice per week, or the combination of GEM and dasatinib (Dasa, 30 mg/kg) was administered by oral gavage four times per week for 2 weeks. Mouse bioluminescent signals were detected on the indicated days after tumor injection. The exposure times for images on the indicated days are indicated. **(H)** Quantitative analysis of relative bioluminescent intensities (mean ± SEM, n = 6 mice) from intrahepatic ICKP tumors, expressed as fold-changes relative to the baseline (Day 3). *, P < 0.05; **, P < 0.005 by two-way ANOVA. NS, non-significant. **(I)** The percentages of CD4⁺ (CD3⁺CD4⁺) T cells, CD8⁺ (CD3⁺CD8⁺) T cells and regulatory T cells (CD4⁺CD25⁺Foxp3⁺) in tumors from panel **(H)**

To clarify the major GEM-metabolic effector in the VTCN1-Src axis, the relative mRNA expressions of GEM metabolic enzymes were evaluated in two pairs of GR cells. Among those genes, the mRNA expressions of *ABCG2* (ATP-binding cassette subfamily G member 2), *NT5C3A* (Cytosolic 5'-nucleotidase 3, NT5C3), and *RRM1* (ribonucleotide reductase catalytic subunit M1) were upregulated, and the protein expressions of RRM1 and NT5C3 were also upregulated in both GR cells (Figure 7A-B). To elucidate how the VTCN1-Src axis regulates the three proteins, VTCN1 and Src expression were manipulated. VTCN1 overexpression induced NT5C3 upregulation in two CCA cells. Moreover, Src overexpression promoted NT5C3 and ABCG2 expression, and NT5C3 was reduced by Src depletion in iCCA cells (Figure 7C-D). In dCK activity, we detected dCK Ser-74 phosphorylation levels to reflect dCK activity 37, and dCK Ser-74 phosphorylation was not modulated by VTCN1 overexpression in both SSP-25 and HuCCT1 cells (Fig. S7A). Src can reduce hENT1 protein expression to mediate resistance in urothelial carcinoma 38, while hENT1 protein levels were not regulated by VTCN1 overexpression in iCCA cells (Fig. S7A). Those results suggest that the VTCN1-Src axis mediates GEM resistance by increasing NT5C3 expression, but not hENT1 expression or dCK activity.

### *In vivo* validation of the VTCN1-Src axis

Finally, we extended our findings *in vivo* by three *in vivo* models to examine how the VTCN1-Src axis drives GEM resistance. In a TAA-induced spontaneous iCCA rat model 25, we first used low-dose GEM (25 mg/kg, once per week for 8 weeks) as initial treatment to establish GEM-resistant iCCA *in vivo*. Subsequently, the rats were divided into three groups. In the previous report, the combination of chemotherapy with an immune checkpoint inhibitor (ICI) is better than ICI alone 39. Thus, the rats continuously received a combined full-dose GEM (50 mg/kg) and an isotype control, a combined full-dose GEM and anti-VTCN1 antibody, or the corresponding vehicles for 4 weeks (Figure 7E). The iCCA tumor sizes in rats were measured using an animal positron emission tomography (*PET*) system 40. In the baseline (1^st^) PET, the relative SUV intensities were undifferentiated in the three groups. At 2 and 4 weeks, rats receiving GEM plus anti-VTCN1 antibody showed significantly reduced relative SUV intensities compared to GEM plus isotype control (2 weeks: P = 0.0277; 4 weeks: P = 0.008, Figure 7F). Tumor-bearing rats receiving the combination of GEM and an isotype control exhibited increased relative SUV intensities, whereas these intensities were significantly reduced in rats treated with the combination of GEM and an anti-VTCN1 antibody (Figure S7B). To confirm the pharmacodynamic effects of anti-VTCN1 antibody treatment *in vivo*, we analyzed the phosphorylated levels of the related proteins. The treatment of anti-VTCN1 antibody and GEM combination reduced Src phosphorylation compared to GEM plus isotype control (Figure S7C). Within the tumor microenvironment, the combination of GEM and anti-VTCN1 antibody significantly increased CD8^+^ T cell infiltration in rat tumor tissues compared to the GEM plus isotype control group (Figure S7D).

In a mouse xenograft model, HuCCT1-GR cells were subcutaneously injected into BALB/c nude mice. Once tumor volumes reached 100 to 150 mm³, the mice were given drugs for 25 days. Dasatinib enhanced GEM-mediated the suppression of tumor growth. Crucially, the combination therapy markedly suppressed tumor growth compared to either monotherapy (Figure S7E-G). In the pharmacodynamic effects, GEM treatment induced the upregulation of the Src protein, leading to an increased amount of Src phosphorylation, and those increases were suppressed by dasatinib treatment. The treatment of dasatinib alone did not reduce Src phosphorylation, which may result from the baseline of Src phosphorylation being low (Figure S7H).

Finally, we experimented with an orthotopic mouse model. Mouse iCCA ICKP cells overexpressing VTCN1 or a control vector 26 were injected into the left lobes of the livers of C57BL/6J mice. The mice were given GEM alone or the combination of GEM and dasatinib for 2 weeks. According to the intensity from bioluminescence images, GEM treatment suppressed tumor growth in control ICKP cells, while GEM treatment did not significantly reduce tumor growth in VTCN1 overexpressing ICKP cells. The combined treatment of GEM and dasatinib significantly suppressed tumor growth in VTCN1 overexpressing ICKP cells *in vivo* (Figure 7G-H). Regarding the tumor microenvironment, VTCN1 overexpression signifi-cantly suppressed CD8^+^ T cell infiltration, whereas it had no discernible effect on the infiltration of CD4^+^ T cells or regulatory T (Treg) cells. The combined treatment of GEM and dasatinib increased CD8^+^ T cell infiltration and decreased Treg cell infiltration (Figure 7I).

**Figure 8:**
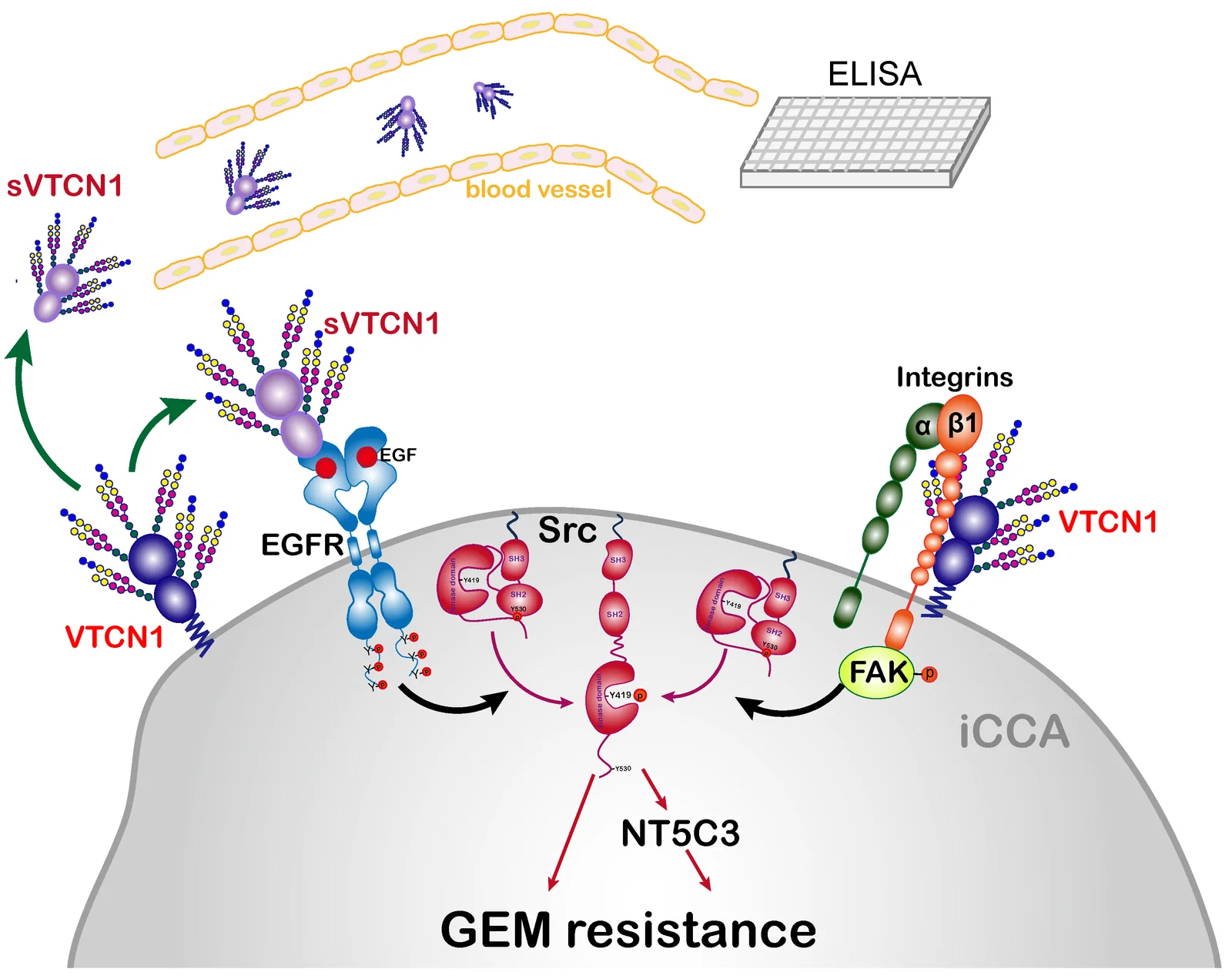
** A diagram illustrates the mechanisms of VTCN1-promoted GR in iCCA.** In iCCA, membrane-bound VTCN1 interacts with integrin β1, which enhances the recruitment and phosphorylation of FAK. Additionally, soluble VTCN1 (sVTCN1) initiates a positive feedback loop by binding to EGFR and promoting its phosphorylation. Both signals lead to Src phosphorylation, and the VTCN1-Src axis promotes NT5C3 expression, contributing to GEM resistance in iCCA. EGFR, epidermal growth factor receptor; ELISA, enzyme-linked immunosorbent assay; FAK, focal adhesion kinase; GEM, gemcitabine; iCCA, intrahepatic cholangiocarcinoma; VTCN1, V-set domain-containing T-cell activation inhibitor 1; NT5C3, cytosolic 5'-nucleotidase 3.

Finally, we summarize this study in Figure 8. At the plasma membrane, membrane-bound VTCN1 is associated with integrin β1 to improve the recruitment and phosphorylation of FAK. Soluble VTCN1 (sVTCN1) induces a positive feedback loop to bind and promote EGFR phosphorylation in iCCA cells. Both signals promote Src phosphorylation, and the VTCN1-Src axis enhances NT5C3 expression, resulting in GEM resistance in iCCA.

## Discussion

Gemcitabine resistance (GR) remains a major challenge for patients with advanced intrahepatic cholangiocarcinoma (iCCA). Here, we demonstrate that VTCN1 contributes to GR in iCCA by promoting integrin β1-FAK interaction and creating a positive feedback loop that activates EGFR. These signaling events collectively drive Src-mediated GR. In the present study, VTCN1 acts as an immunosuppressor to promote tumor progression 19. Interestingly, our findings reveal a previously unrecognized role of soluble VTCN1 (sVTCN1) in cancers. Beyond serving as a prognostic or predictive marker, sVTCN1 functions as an autocrine regulator by binding to and activating EGFR. While the receptor for VTCN1 remains unidentified, our study provides evidence that VTCN1-mediated cell functions occur in a cell-cell contact-independent manner (Figure 8).

Two GEM-resistant mechanisms are identified: Intrinsic (primary) resistance and acquired (secondary) resistance 41. In our study, the increased VTCN1 expression was detected after 72 h of GEM exposure, and the increased expression was not detected after 2 weeks of GEM exposure in iCCA. The transient VTCN1 upregulation may result from GEM-induced transient cell stress, promoting the activation of STAT3 42, 43. For long-term exposure (2 months to 4 months), VTCN1 expression was sustainably upregulated. VTCN1 knockdown in GR sublines partially increased GEM sensitivity but did not fully revert to parental sensitivity levels (Figure 1). Thus, the function of VTCN1 appears to contribute mainly to acquired resistance.

In our cohort, high VTCN1 expression was positively associated with shorter PFS, but not OS. A similar pattern is found in other studies 44, 45. PFS immediately reflects the current therapeutic strategy, while OS is influenced by multiple medical interventions. In future studies, VTCN1 may participate in evaluating or predicting resistance to other chemotherapies, target therapies, or immunotherapies.

Soluble VTCN1 (sVTCN1, sB7H4) has been determined in several cancer patients 20, 22, 46. Despite these clinical correlations, no studies have previously elucidated the mechanism of sVTCN1-mediated tumor progression. Here, we identified a new concept for VTCN1 in B7 family biology. We first demonstrated that soluble B7 family protein binds to EGFR, triggering a positive feedback loop that activates Src. sVTCN1-EGFR interaction provided several clinical implications: (1) Circulating sVTCN1 can systemically promote GEM resistance and EGFR activation; (2) sVTCN1 enhances EGFR signaling on neighboring cells via a paracrine loop; (3) The levels of sVTCN1 correlated with tumor burden (Figure 2F-H), acting as a complement biomarker to monitor treatment effects; (4) sVTCN1-EGFR axis provides an option for a combined treatment of VTCN1 antibody (or antibody drug conjugate, ADC) and EGFR inhibitors in iCCA patients with high VTCN1 and EGFR expression. Future studies should focus on whether the VTCN1 antibody (or ADC) neutralizes sVTCN1 and membrane VTCN1 to improve anti-cancer effects.

Previous studies have explored the interplay between integrins and another B7 family protein, PD-L1 47, 48. Notably, our study was the first to demonstrate a direct interaction between an integrin and a B7 family protein. Our results demonstrated that VTCN1 can enhance Src phosphorylation (Figure 3), but does not directly bind Src (Figure 4B). Instead of a direct interaction, VTCN1 activated Src through two mediators, EGFR and integrin β1. Membrane VTCN1 was directly associated with integrin β1, enhancing FAK recruitment and phosphorylation (Figure 4), and sVTCN1 directly bound EGFR, promoting EGF-induced autophosphorylation (Figure 5). Both signals contributed to Src activation. In the rescue experiments, Src overexpression could compensate for the decreased GEM IC_50_ values caused by VTCN1 depletion, and Src family inhibitor dasatinib treatment impaired the increased GEM IC50 values induced by Src and VTCN1 overexpression (Figure 3), suggesting Src expression and activity are sufficient for VTCN1-mediated GEM resistance. The dual-targeting of VTCN1 and integrin β1 facilitated an advantageous effect in decreasing GEM IC_50_ values compared to VTCN1 or integrin β1 alone (Figure 4K-L). Given these findings, the development of a dual-targeting antibody against VTCN1 and an integrin β1 antibody (antagonist) could provide a novel therapeutic strategy for iCCA or other cancers in which VTCN1-integrin β1 interactions play a role.

Several limitations of our study should be acknowledged. First, although we demonstrated that Src inhibition overcomes GR in preclinical models, optimal clinical dosing and patient selection criteria require investigation. A randomized phase II trial shows that there is no survival benefit when dasatinib is added to GEM in advanced pancreatic cancer 49. Second, the protease for generating sVTCN1 in iCCA from membrane-bound VTCN1 remains unidentified, representing an area for future investigation. Third, we focus on GEM resistance; whether VTCN1-Src activation contributes to resistance against other iCCA-relevant therapies requires study. Fourth, CA19-9 and CEA showed no significant association with PFS in univariate analysis (Table 1). Moreover, hENT1 and dCK status were detected in cell lines *in vitro* and were not stained in the iCCA clinical specimens; the related results cannot be provided in this manuscript. Fifth, though we identified that NT5C3 is the effector of the VTCN1-Src axis to mediate GEM metabolism, we did not detect the levels of dFdCTP or the ratios of dC/dCTP. Thus, the association of the VTCN1-Src axis and GEM metabolism remains to be clarified.

## Conclusions

In summary, this study uncovers a novel role of VTCN1 in GR iCCA. We demonstrate that membrane-bound VTCN1 interacts with integrin β1, as well as soluble VTCN1 (sVTCN1), which binds to EGFR, contributing to increased FAK and EGFR phosphorylation. These interactions collectively drive Src-mediated GR. sVTCN1 binds EGFR and enhances EGF-induced receptor activation, extending the current understanding of B7 family proteins. Importantly, we first identify the potential prognostic and predictive value of VTCN1 and propose a dual-targeting strategy against VTCN1 and integrin β1 as a promising therapeutic approach to overcome GR in iCCA.

## Supplementary Material

Supplementary figures.

Supplementary tables.

## Figures and Tables

**Table 1:** Univariate analysis of prognostic factors regarding progression-free survival in patients with stage III-IV iCCA.

Parameter	Total N	N of events (%)	Median (months)	95% CI of median	P-Value
Gender					0.503
Male	35	23 (65.7)	3.35	1.99 - 4.72	
Female	37	32 (86.5)	5.65	4.34 - 6.96	
Age (years)					0.843
< 65	44	34 (77.3)	5.4	3.2 - 7.6	
≥ 65	28	21 (75.0)	4.3	1.2 - 7.3	
CEA (ng/mL)					0.207
< 5	34	26 (76.5)	5.7	5.1 - 6.2	
≥ 5	23	18 (78.3)	2.9	2.6 - 3.2	
Unknown	15	11 (73.3)	5.8	2.2 - 9.5	
CA19-9 (U/L)					0.796
< 37	18	14 (77.8)	3.1	1.7 - 4.5	
≥ 37	40	31 (77.5)	5.8	3.8 - 7.8	
Unknown	14	10 (71.4)	3.6	0.3 - 6.9	
Albumin (g/dL)					0.840
≤ 3.5	17	11 (64.7)	5.8	1.7 - 9.9	
> 3.5	40	32 (80.0)	3.8	2.9 - 4.8	
Unknown	15	12 (80.0)	5.4	1.9 - 8.9	
Total bilirubin (mg/dL)					0.634
≤ 1.3	52	43 (82.7)	4.8	2.9 - 6.8	
> 1.3	13	7 (53.8)	6.5	0.2 - 12.8	
Unknown	7	5 (71.4)	2.9	1.8 - 4.0	
H-score of VTCN1					**< 0.0001**
≤ 65	52	36 (69.2)	5.8	4.5 - 7.1	
> 65	20	19 (95.0)	2.7	2.4 - 2.9	
Viral hepatitis					0.149
None	44	34 (77.3)	4.0	1.8 - 6.3	
B or C	28	21 (75.0)	5.8	2.9 - 8.8	
Tumor no.					**0.011**
Single	35	25 (71.4)	5.8	4.9 - 6.8	
Multiple	37	30 (81.1)	3.3	2.3 - 4.2	
Tumor size (cm)					0.408
≤ 5	12	7 (58.3)	4.3	3.6 - 5.0	
> 5	51	42 (82.4)	3.6	1.4 - 5.8	
Unknown	9	6 (66.7)	6.5	4.8 - 8.3	
Stage					0.983
III	10	8 (80.0)	5.39	4.75 - 6.03	
IV	62	47 (75.8)	4.04	1.74 - 6.35	
Chemotherapy					0.202
GEM	10	9 (90.0)	3.35	1.17 - 5.54	
GEM + CIS	62	46 (74.2)	5.39	3.43 - 7.35	
Best response					**< 0.0001**
PR	10	7 (70.0)	11.1	9.0–13.3	
SD	32	18 (56.2)	7.0	5.1–8.9	
PD	30	30 (100)	2.6	2.2–2.9	

Abbreviations: CI, confidence interval; CEA, carcinoembryonic antigen; CA19-9, carbohydrate antigen 19-9; HBV, hepatitis B virus; HCV, hepatitis C virus; GEM, gemcitabine; CIS, cisplatin.

## Data Availability

The cDNA microarray datasets for SSP-25 and SSP-25-GR cells, alongside the RNA sequencing datasets for various CCA cells (SSP-25, SSP-25-GR, KKU213, and KKU213-GR), were deposited in the Gene Expression Omnibus (GEO) database under accession numbers GSE291223 and GSE292343, respectively.

## Abbreviations

CM: conditioned media
CI: combination index
EGFR: epidermal growth factor receptor
GEM: gemcitabine
GR: gemcitabine resistance
HR: hazard ratio
IHC: immunohistochemistry
iCCA: intrahepatic cholangiocarcinoma
FAK: focal adhesion kinase
NT5C3: cytosolic 5'-nucleotidase 3
OS: overall survival
PET: positron emission tomography
PFS: progression-free survival
PLA: proximity ligation assay
PD: progressive disease
PR: partial response
SD: stable disease
sVTCN1: soluble v-set domain containing T cell activation inhibitor 1
RT-qPCR: reverse transcription quantitative polymerase chain reaction
SUV: standardized uptake value
TAA: thioacetamide
WB: western blot
