# Supplementary material for: Membrane and soluble VTCN1 (B7H4) converge on Src signaling to mediate gemcitabine resistance in intrahepatic cholangiocarcinoma
Source: Theranostics. 2026 Aug 24;16(15):8968–86. doi: 10.7150/thno.130394 (PMC13549373; doi:10.7150/thno.130394)
Supplement: Supplementary file 1 [file thnov16p8968s1.pdf]

Supplementary Figures

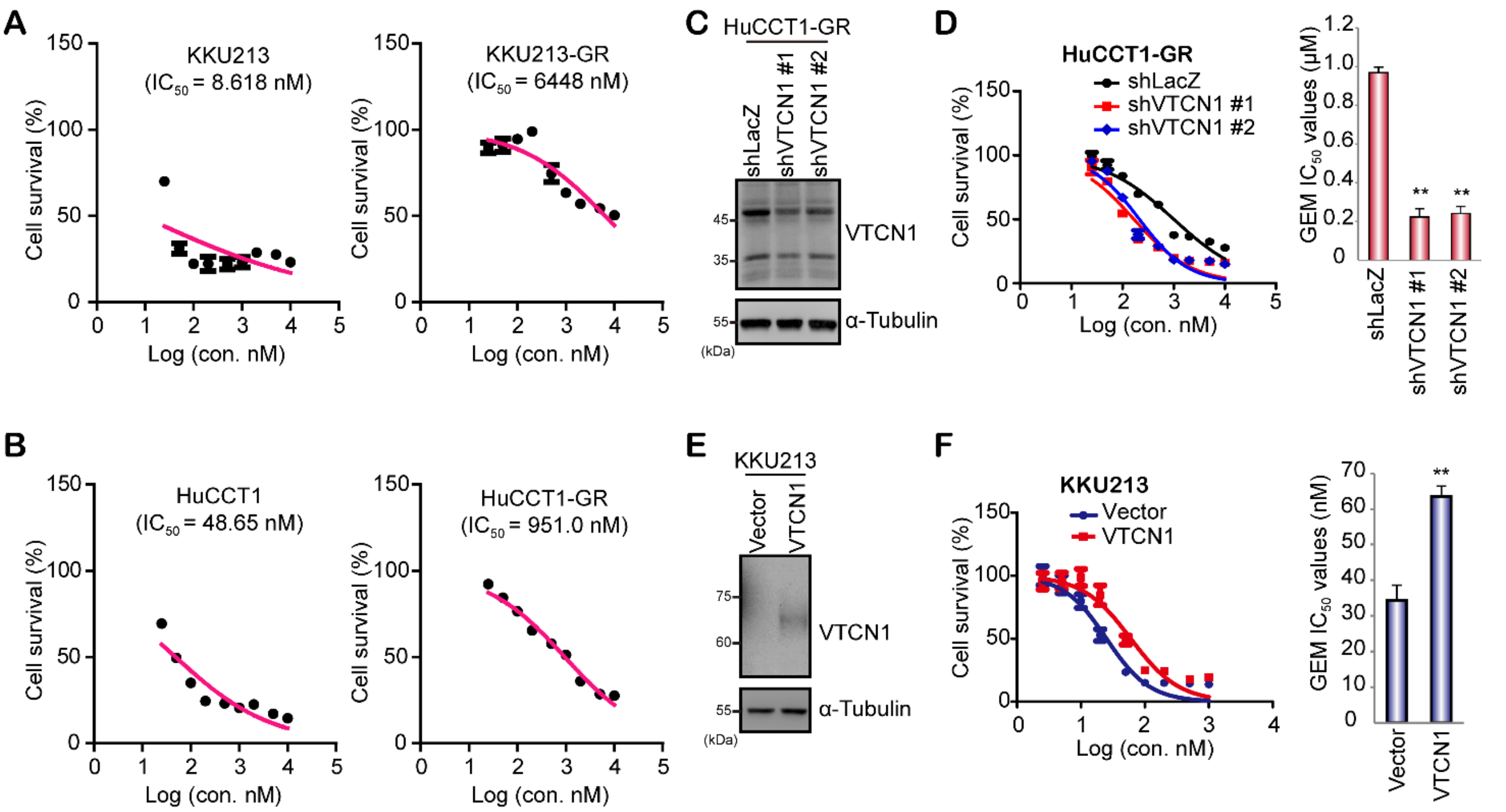

Supplementary Figure 1. VTCN1 expression is increased in iCCA cells with GEM resistance.

(A) Cell viability curves of KKU213 and KKU213-GR cells across various GEM concentrations, with the corresponding IC_{50} values indicated in the panel (mean ± SD, N = 3).

(B) Cell viability curves of HuCCT1 and HuCCT1-GR cells across various GEM concentrations, with the corresponding IC_{50} values indicated in the panel (mean ± SD, N = 3).

(C) WB displaying the protein levels of indicated targets in HuCCT1-GR cells expressing shRNA targeting VTCN1 (shVTCN1) or a control sequence (shLacZ).

(D) Cell viability curves (left) and GEM IC_{50} values (right; mean ± SD, N = 3) in HuCCT1-GR cells transfected with shRNA targeting VTCN1 (shVTCN1) or a control sequence (shLacZ).

(E) WB displaying the protein levels of indicated targets in KKU213 cells overexpressing FLAG-tagged VTCN1 (VTCN1) or a control vector (Vector).

(F) Cell viability curves (left) and GEM IC_{50} values (right; mean ± SD, N=3) in KKU213 cells overexpressing FLAG-tagged VTCN1 (VTCN1) or a control vector (Vector).

**, P < 0.005 by Student's *t*-test.

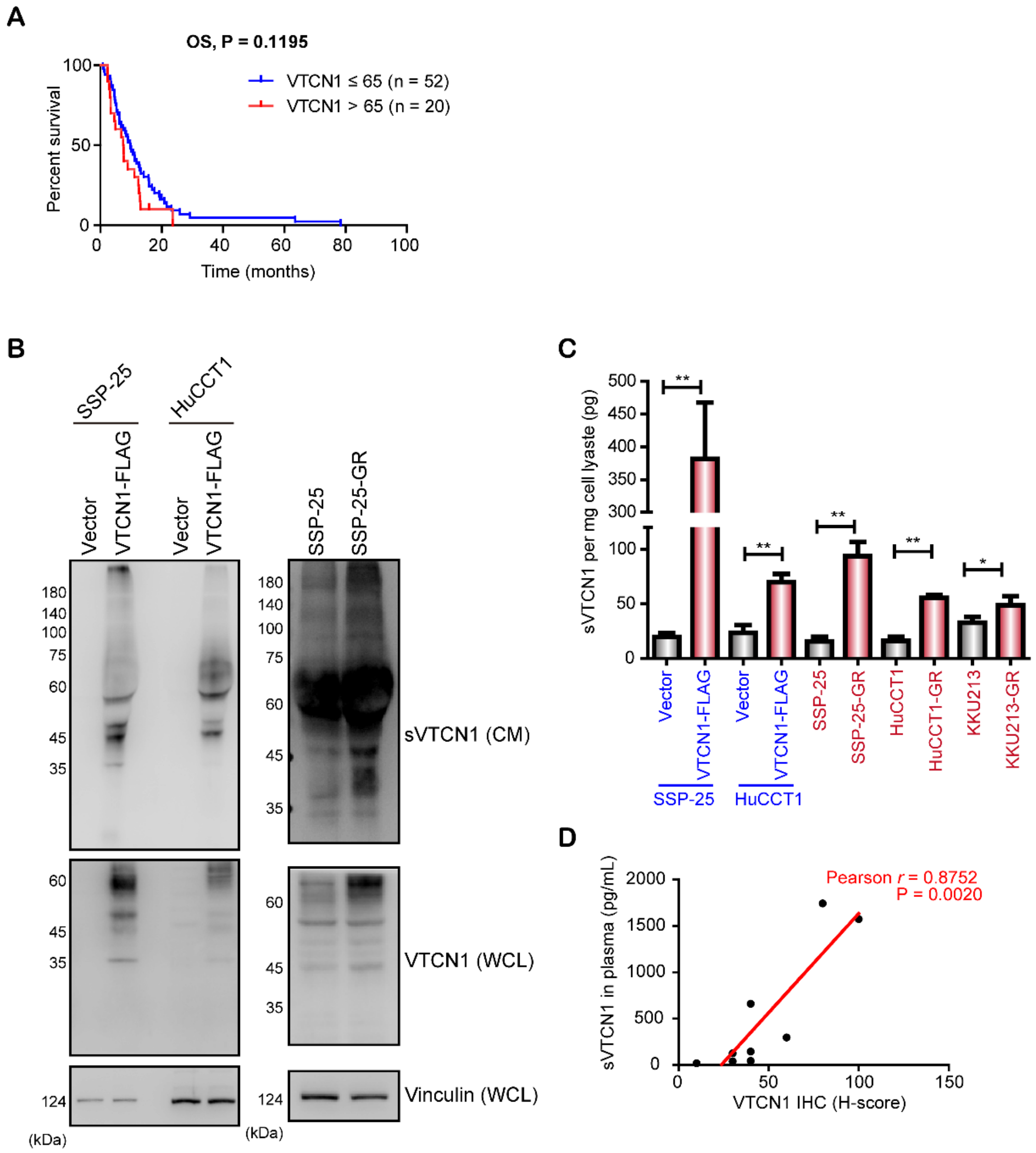

Supplementary Figure 2. Soluble VTCN1 (sVTCN1) is detected in the conditioned media from iCCA cells.

(A) Kaplan-Meier plot of the overall survival (OS) of iCCA patients with low VTCN1 expression (VTCN1 ≤ 65, blue) or high VTCN1 expression (VTCN1 > 65, red). Statistical significance (P-value) was assessed using the log-rank test.

(B) Left: WB showing the protein levels of indicated targets from whole cell lysates (WCL) and soluble VTCN1 (sVTCN1) from concentrated conditioned media (CM) in SSP-25 or HuCCT1 cells overexpressing FLAG-tagged VTCN1 (FLAG-VTCN1) or the control vector (Vector). Right: WB showing the protein levels of indicated targets from whole cell lysates (WCL) and soluble VTCN1 (sVTCN1) from concentrated conditioned media in SSP-25 cells or SSP-25-GR cells.

(C) The amount of soluble VTCN1 (sVTCN1) in conditioned media from SSP-25, SSP-25GR, KKU213, KKU213-GR, HuCCT1, HuCCT1-GR or the indicated cells overexpressing FLAG-tagged VTCN1 (VTCN1-FLAG) or the control vector (Vector). The values (mean ± SD) were from three independent experiments. **, $P < 0.005$ and *, $P < 0.05$ (Student's *t*-test).

(D) Correlation analysis between VTCN1 protein expression in formalin-fixed paraffin-embedded samples and soluble VTCN1 (sVTCN1) in the blood plasma samples from nine iCCA patients. The panel displays the Pearson correlation coefficient (*r*) and the corresponding P values.

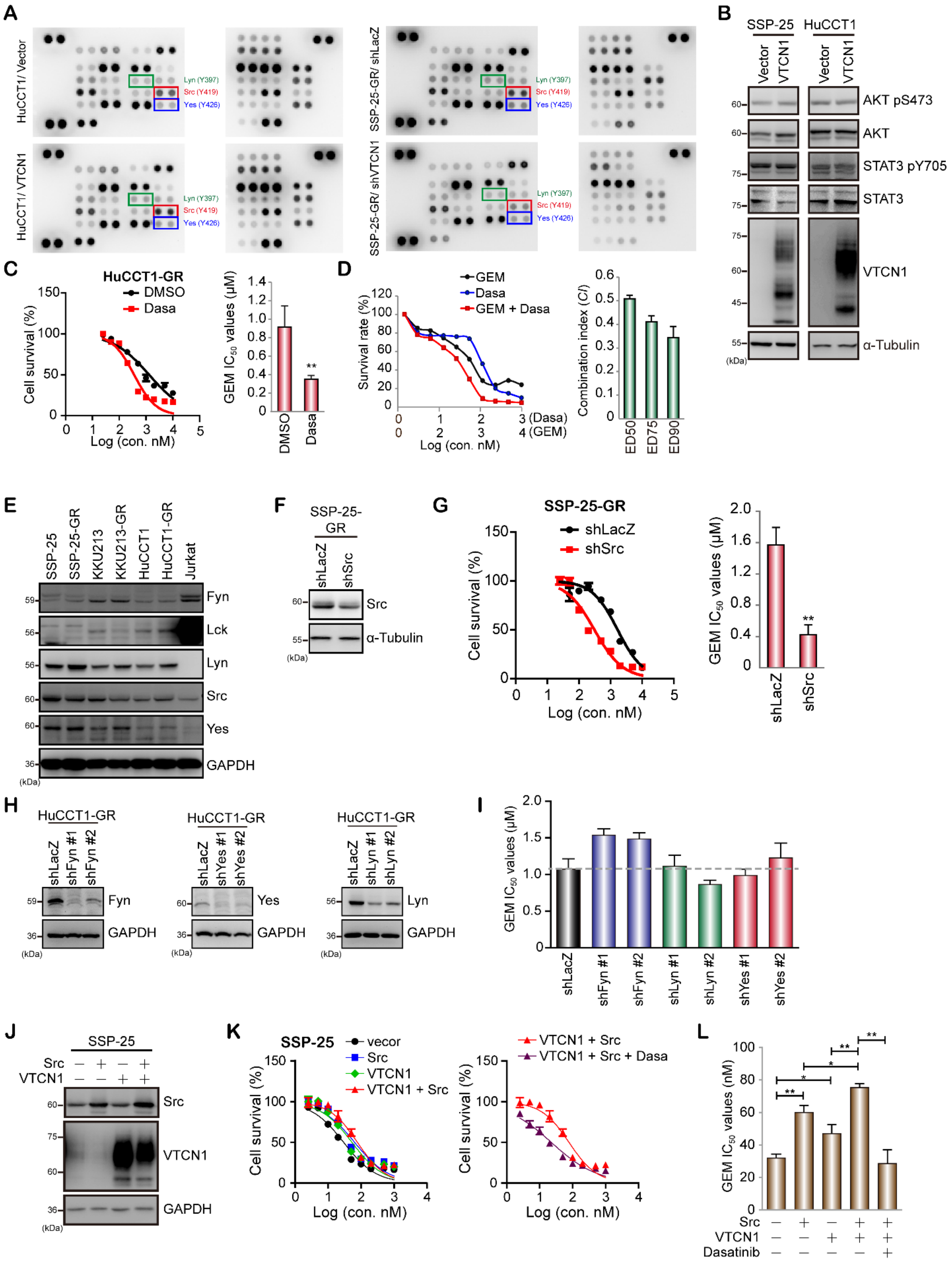

Supplementary Figure 3. Src is involved in VTCN1-mediated GR in iCCA.

(A) Whole-cell lysates were prepared from HuCCT1 cells overexpressing VTCN1 (HuCCT1/ VTCN1) or a control vector (HuCCT1/ Vector) and SSP-25-GR cells transfected with shRNA

targeting VTCN1 (SSP-25-GR/ shVTCN1) or a control sequence (SSP-25-GR/ shLacZ) and hybridized the nitrocellulose membranes with 39 antibodies from a phospho-kinase array kit (ARY003C, R&D). Red lines, blue lines, and green lines indicate the phosphorylation of Src, Yes, and Lyn.

(B) WB displaying the protein levels of indicated targets in the cells overexpressing FLAG-tagged VTCN1 (VTCN1) or a control vector (Vector).

(C) Left: Cell viability curves of HuCCT1-GR cells subjected to varying concentrations of GEM in the presence of DMSO or 50 nM dasatinib (Dasa) for 72 h. Right: Corresponding GEM IC_{50} values (mean ± SD, N = 3).

(D) Left: Cell viability curves of HuCCT1-GR cells subjected to varying concentrations of dasatinib (Dasa) and GEM for 72 h. Right: Combination index (*CI*) values (mean ± SD, N = 3) in the cells. ED, effective dose.

(E) WB displaying the protein levels of indicated targets in iCCA cell lines and Jurkat cells.

(F) WB displaying the protein levels of indicated targets in the cells transfected with shRNA targeting Src (shSrc) or a control sequence (shLacZ).

(G) Cell viability curves (left) and GEM IC_{50} values (right; mean ± SD, N = 3) in SSP-25-GR cells transfected with shRNA targeting Src (shSrc) or a control sequence (shLacZ).

(H) WB showing the protein levels of indicated targets in HuCCT1-GR cells transfected with shRNA targeting Fyn (shFyn), Lyn (shLyn), Yes (shYes), or a control sequence (shLacZ).

(I) GEM IC_{50} values (mean ± SD, N = 3) in HuCCT1-GR cells transfected with shRNA targeting Fyn (shFyn), Lyn (shLyn), Yes (shYes), or a control sequence (shLacZ).

(J) WB displaying the protein levels of indicated targets in VTCN1- overexpressed (VTCN1, +) SSP-25 cells receiving Src overexpression (Src, +) or the empty control vector (Src, -).

(K) Cell viability curves in VTCN1- overexpressed SSP-25 cells receiving Src overexpression or the empty control vector (left) and in the presence of DMSO or 10 nM dasatinib (Dasa; right) for 72 h in SSP-25-overexpressing VTCN1 and Src cells.

(L) GEM IC_{50} values (right; mean ± SD, N = 3) in VTCN1- overexpressed SSP-25 cells receiving Src overexpression (Src, +) or a control vector (Src, -) and in the presence of DMSO (Dasa, -) or 10 nM dasatinib (Dasa, +) for 72 h in SSP-25-overexpressing VTCN1 and Src cells.

** $P < 0.005$; *, $P < 0.05$ by Student's *t*-test. NS, non-significant.

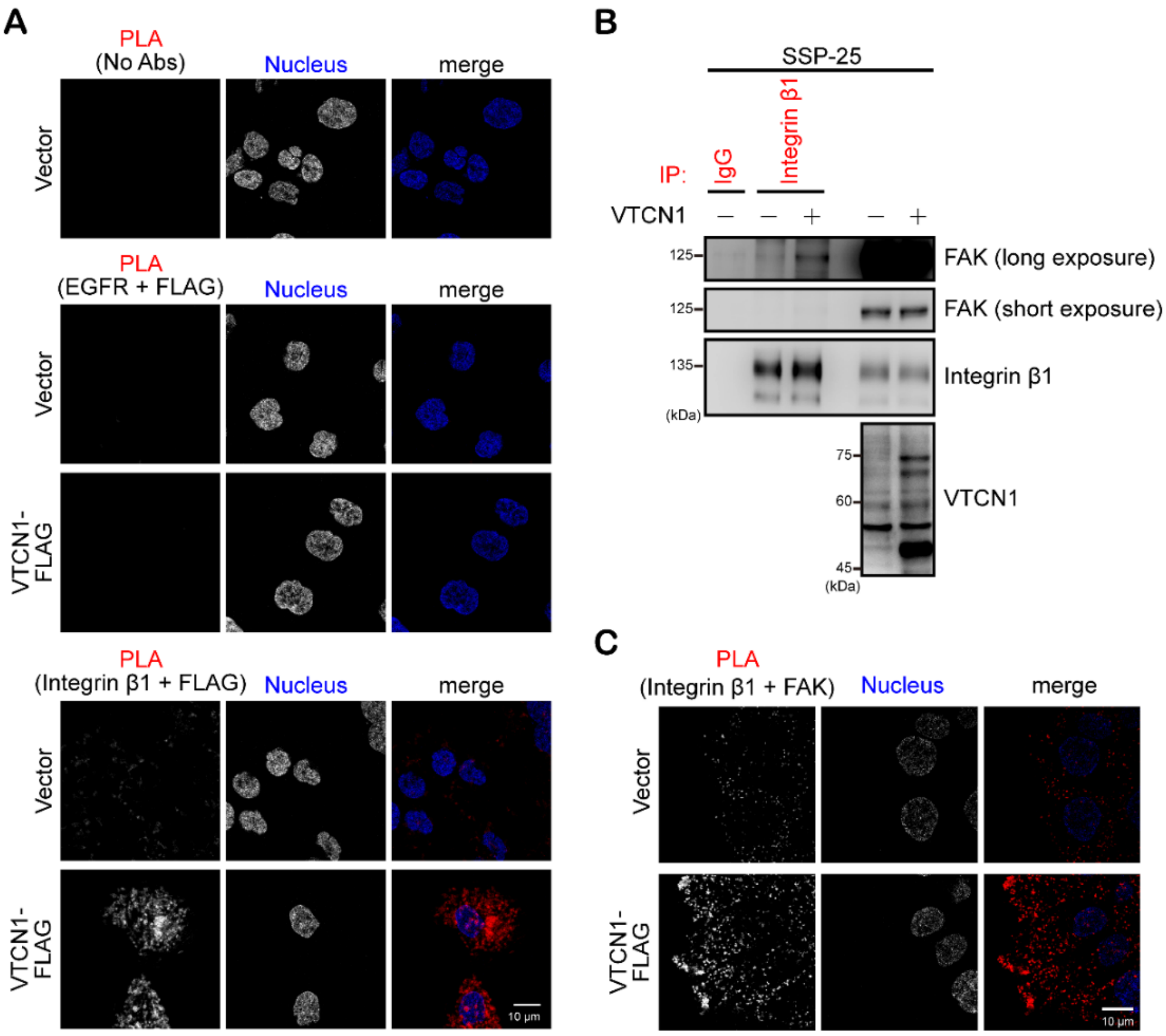

Supplementary Figure 4. VTCN1 interacts with integrin β1 subunit.

(A) A duolink proximity ligation assay (PLA) showing the interactions between FLAG-tagged VTCN1 (VTCN1-FLAG) and integrin β1 or EGFR in situ. SSP-25 cells expressing FLAG-tagged VTCN1 or a control vector (Vector) and were grown on coverslips overnight. A duolink assay was performed and DNA was stained with DAPI (blue). The red spots in PLA indicated integrin β1 and FLAG-tagged VTCN1 interaction.

(B) Immunoprecipitation-western blot showing the effect of VTCN overexpression on integrin β1 and FAK interaction. FLAG-tagged VTCN1 (+) or a control vector (-) was transiently overexpressed in SSP-25-GR cells. The immunocomplexes, immunoprecipitated (IP) using an integrin β1 antibody or control IgG, and whole-cell lysates were analyzed with the indicated antibodies.

(C) A duolink proximity ligation assay (PLA) showing the effect of VTCN overexpression on integrin β1 and FAK interaction in situ. KKU-213 cells expressing FLAG-tagged VTCN1 or a control vector (Vector) and were grown on coverslips overnight. A duolink assay was performed and DNA was stained with DAPI (blue). The red spots in PLA indicated integrin β1 and FAK interaction.

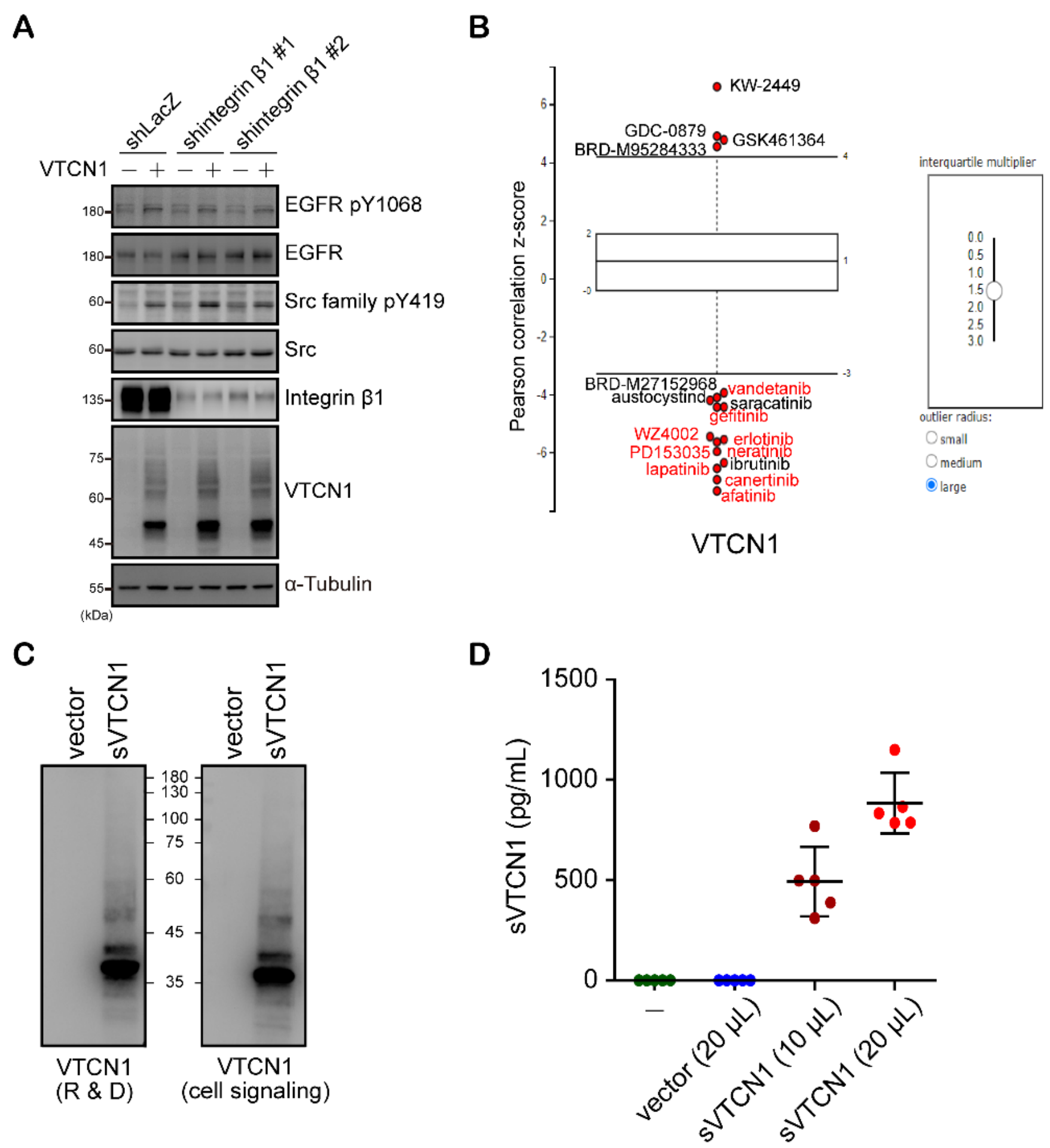

Supplementary Figure 5. sVTCN1 interacts with EGFR.

(A) WB displaying the protein levels of indicated targets in the SSP-25-GR subline. The cells were transduced with shRNA against integrin β1 (shIntegrin β1 #1 and #2) or the empty control sequence (shLacZ), and then FLAG-tagged VTCN1 (VTCN1, +) or the control vector (VTCN1, -) was transiently overexpressed.

(B) Correlation between the IC_{50} of small-molecule compounds and the mRNA level of VTCN1 was calculated using the Cancer Therapeutics Response Portal (CTRP, https://portals.broadinstitute.org/ctrp). Red indicates HER family-related inhibitors/TKIs. Interquartile range (IQR) = 1.5.

(C) WB showing 1 μL purified sVTCN1 from HEK293 cell-derived CM. HEK293 cells were transiently expressing FLAG-tagged VTCN1 or the control vector (Vector) for 24 h and then replaced with serum-free media for another 24 h. sVTCN1 was purified from serum-free CM by FLAG beads and eluted with 3x DYKDDDDK. sVTCN1 proteins were detected by two

commercial anti-VTCN1 antibodies (Table S5).

(D) The concentrations of 20 μL sVTCN1 protein (sVTCN1), 10 μL sVTCN1 protein (sVTCN1), or the control vector (vector) in 1 mL RPMI were detected using the VTCN1 ELISA kit. The concentrations from the five purifications were determined. －, RPMI alone.

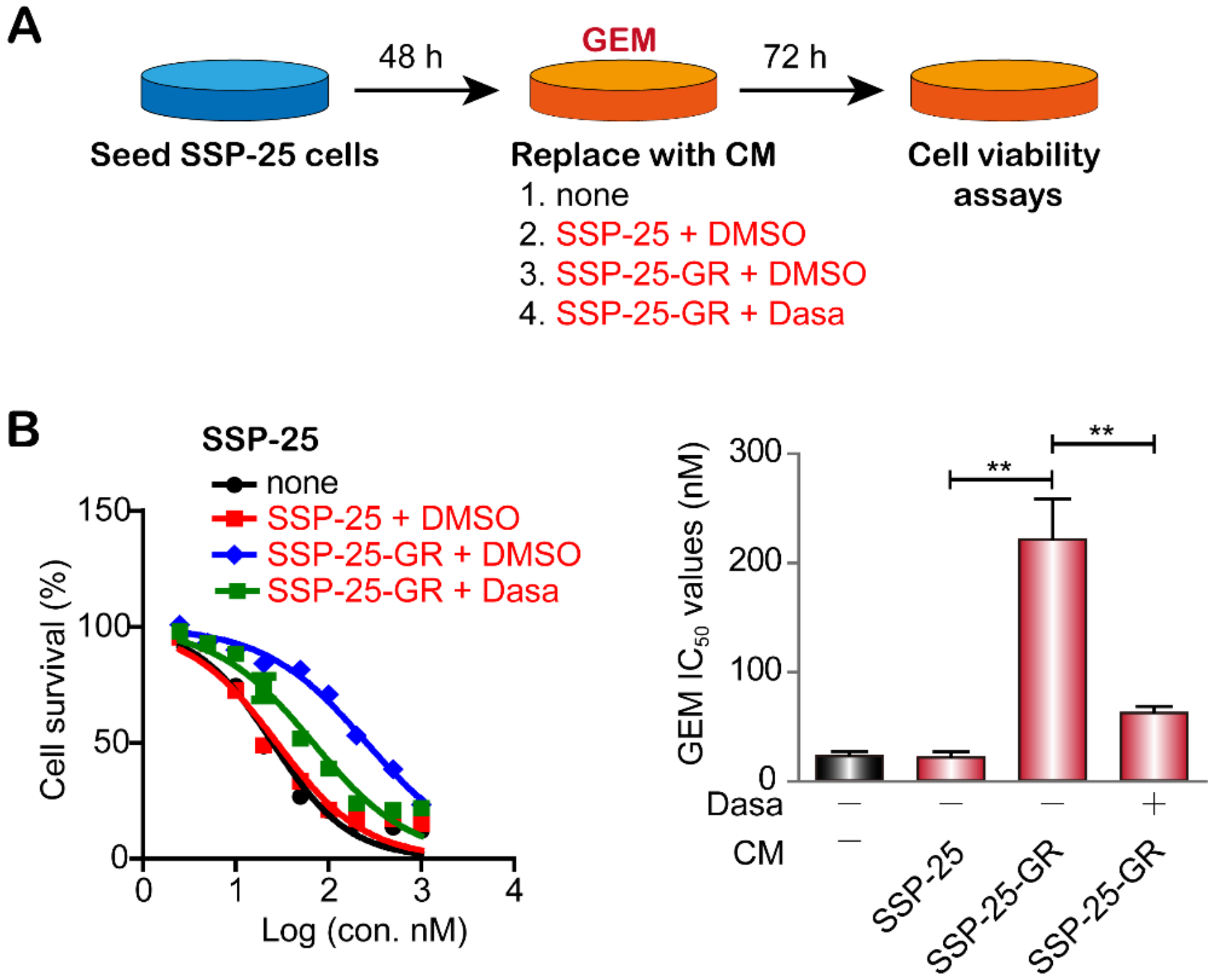

Supplementary Figure 6. Dasatinib treatment impairs SSP-25-GR-derived CM-upregulated GEM IC_{50} values.

(A) A schema for the experimental design for the cell viability assay in panel B. The conditioned media (CM) were collected from SSP-25 cells or SSP-25-GR cells. SSP-25 cells were grown for 48 h and then treated with various concentrations of GEM alone (none) or the combination of various concentrations of GEM, the CM, and 100 nM dasatinib (Dasa) or DMSO for another 72 h.

(B) Left: Cell viability curves of SSP-25 cells cultured with or without (none) CM, in combination with 100 nM dasatinib (Dasa) or DMSO, across various GEM concentrations. Right: The GEM IC_{50} values (mean ± SD, N = 3) in SSP-25 cells treated with or without (-) the combination of the CM and 100 nM dasatinib (Dasa, +) or DMSO (-). **, $P < 0.005$ by Student's *t*-test.

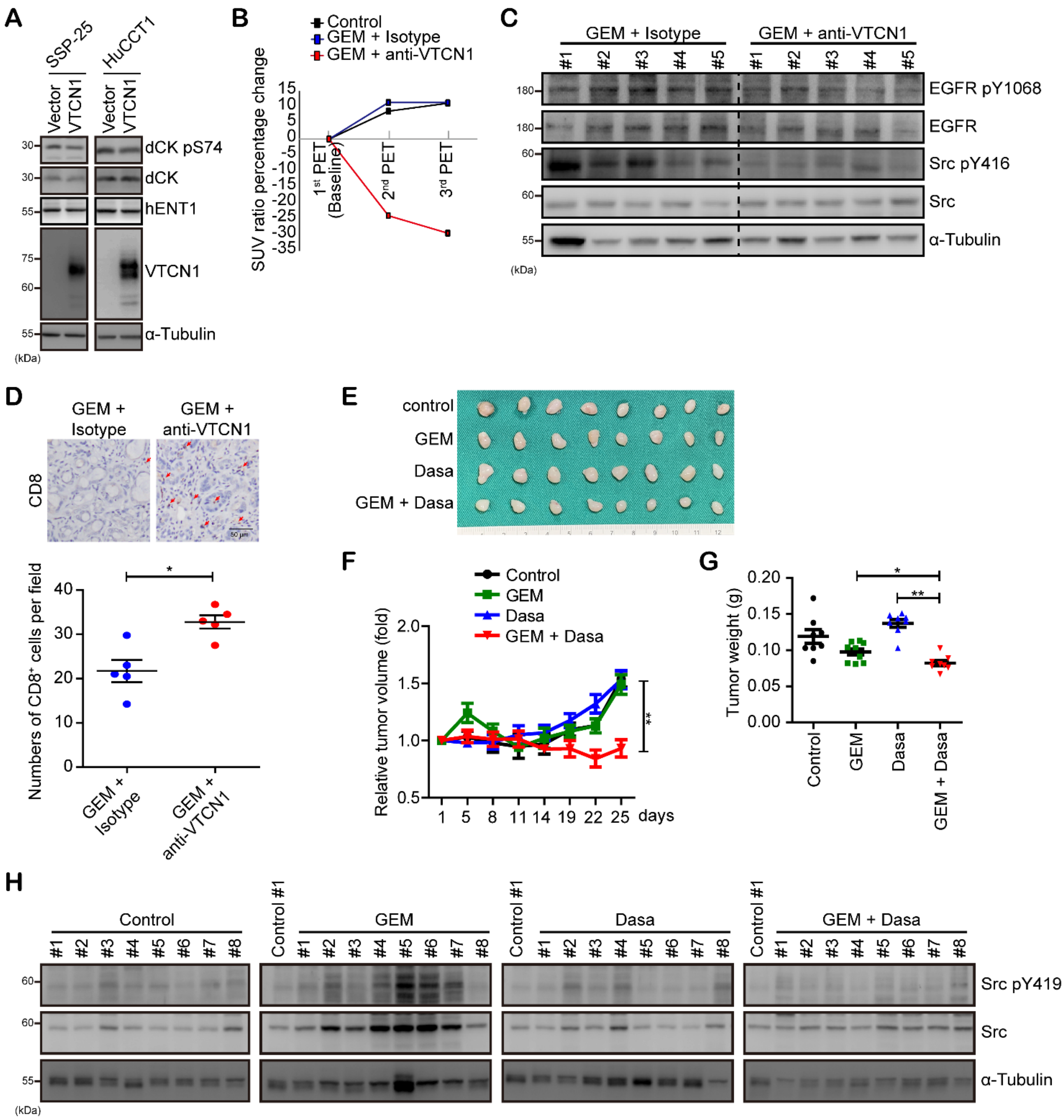

Supplementary Figure 7. Combination therapy of GEM and dasatinib reduces tumor growth in vivo.

(A) WB showing the protein levels of indicated targets in the cells overexpressing VTCN1 or the empty control vector (Vector).

(B) The average change values of the tumor-to-liver (T/L) ratio of SUV in the control and experimental groups at 2 weeks (2nd PET) and 4 weeks (3rd PET) after the experiment.

(C) WB showing the protein levels of indicated targets in iCCA rat tissues receiving the treatment of the GEM and a control isotype combination or GEM and VTCN1 antibody combination.

(D) Upper: Immunohistochemical detection of CD8 in TAA-induced iCCA tissues. Scale bar = 50 μm. Lower: Quantification of $CD8^+$ cells per field in TAA-induced iCCA rats following 8 weeks of low-dose GEM treatment, then GEM + a isotype control antibody (n = 5), or GEM + anti-VTCN1 antibody (n = 5) for 4 weeks. Values presented as mean ± SEM. Red arrows, CD8 positive cells. *, $p < 0.05$ by Student's *t*-test.

(E) Representative images of the xenograft animal model. HuCCT1-GR cells were subcutaneously inoculated into BALB/c nude mice. The mice were given 50 mg/kg GEM alone by intraperitoneal injection once per week, 20 mg/kg dasatinib (Dasa) alone by oral gavage five times per week, or the combination of GEM and dasatinib (Dasa) for 25 days.

(F) Relative tumor volumes from the experiment described in panel (E). Data represent mean ± SEM (n = 8). **, $P < 0.005$ by two-way ANOVA.

(G) The tumor weight of the experiment from panel (E). Values are expressed as mean ± SEM (n = 8). *, $P < 0.05$; **, $P < 0.005$ by Student's *t*-test.

(H) WB displaying the protein levels of indicated targets in CCA tumors from xenograft experiments receiving the treatment of the control solvent, GEM alone, dasatinib (Dasa) alone, or the combination of GEM and dasatinib (GEM + Dasa).
